# Fatty acid metabolism constrains Th9 cell differentiation and antitumor immunity *via* the modulation of retinoic acid receptor signaling

**DOI:** 10.1038/s41423-024-01209-y

**Published:** 2024-08-26

**Authors:** Takahiro Nakajima, Toshio Kanno, Yuki Ueda, Keisuke Miyako, Takeru Endo, Souta Yoshida, Satoru Yokoyama, Hikari K. Asou, Kazuko Yamada, Kazutaka Ikeda, Yosuke Togashi, Yusuke Endo

**Affiliations:** 1 Department of Frontier Research and Development, Laboratory of Medical Omics Research, Kazusa DNA Research Institute, 2-6-7 Kazusa Kamatari, Kisarazu, Chiba 292-0818 Japan; 2 Department of Tumor Microenvironment, Faculty of Medicine, Dentistry and Pharmaceutical Sciences, Okayama University, 2-5-1, Shikata-cho, Kita-ku, Okayama 700-8558 Japan; 3 Department of Applied Genomics, Kazusa DNA Research Institute, 2-6-7 Kazusa Kamatari, Kisarazu, Chiba 292-0818 Japan; 4 Division of Cell Therapy, Chiba Cancer Center Research Institute, 666-2 Nitona-cho, Chuo-ku, Chiba 260-8717 Japan; 5 Department of Omics Medicine, Graduate School of Medicine, Chiba University, 1-8-1 Inohana, Chuo-ku, Chiba 260-8670 Japan

**Keywords:** Th9 cell, immunometabolism, omics analysis, fatty acid, RARα, anti-tumor effect, Interleukins, Tumour immunology, Lipid signalling

## Abstract

T helper 9 (Th9) cells are interleukin 9 (IL-9)-producing cells that have diverse functions ranging from antitumor immune responses to allergic inflammation. Th9 cells differentiate from naïve CD4^+^ T cells in the presence of IL-4 and transforming growth factor-beta (TGF-β); however, our understanding of the molecular basis of their differentiation remains incomplete. Previously, we reported that the differentiation of another subset of TGF-β–driven T helper cells, Th17 cells, is highly dependent on de novo lipid biosynthesis. On the basis of these findings, we hypothesized that lipid metabolism may also be important for Th9 cell differentiation. We therefore investigated the differentiation and function of mouse and human Th9 cells in vitro under conditions of pharmacologically or genetically induced deficiency of the intracellular fatty acid content and in vivo in mice genetically deficient in acetyl-CoA carboxylase 1 (ACC1), an important enzyme for fatty acid biosynthesis. Both the inhibition of de novo fatty acid biosynthesis and the deprivation of environmental lipids augmented differentiation and IL-9 production in mouse and human Th9 cells. Mechanistic studies revealed that the increase in Th9 cell differentiation was mediated by the retinoic acid receptor and the TGF-β–SMAD signaling pathways. Upon adoptive transfer, ACC1-inhibited Th9 cells suppressed tumor growth in murine models of melanoma and adenocarcinoma. Together, our findings highlight a novel role of fatty acid metabolism in controlling the differentiation and in vivo functions of Th9 cells.

## Introduction

T helper 9 (Th9) cells are interleukin 9 (IL-9)-producing cells that differentiate from naïve CD4^+^ T cells in the presence of IL-4 and transforming growth factor-β (TGF-β) [1–3]. Compared with other subsets, such as Th2, Th17, and regulatory T cells, which produce only small amounts of IL-9, Th9 cells appear to constitute a T-cell subset specialized for IL-9 production. However, Th9 cells are closely related to the Th2 cells that are detected in allergic diseases, and increasing evidence has indicated that Th9 cells may have a potent ability to eradicate advanced tumors, particularly melanomas [4]. Moreover, recent reports indicate that tumor-specific Th9 cells are less susceptible to exhaustion, have complete cytolytic activity against tumor cells, and have prolonged persistence capacity because of their distinctive hyperproliferative characteristics [5, 6].

The signaling cascade mediated by TGF-β plays a pivotal role in the differentiation of Th9 cells [7]. The engagement of TGF-β with its cognate receptor activates specific SMAD family members. For example, phospho-SMAD3 directly binds to the *Il9* gene locus, forming a complex with the Notch intracellular domain and recombination signal binding protein for the immunoglobulin kappa J region [2, 8, 9]. Furthermore, TGF-β–activated kinase mitogen-activated protein kinase kinase kinase 7 (MAP3K7, also known as TAK1), a key mediator of the SMAD-independent arm of the TGF-β signaling cascade, controls Th9 cell differentiation through the downregulation of DNA-binding protein inhibitor (ID3) [10, 11]. The SMAD-independent TGF-β signaling pathway also involves other mitogen-activated protein kinases (MAPKs), namely, extracellular signal-regulated kinases 1/2, c-Jun N-terminal kinases, p38 MAPKs, phosphatidylinositol-3-kinase (PI3K)/protein kinase B (Akt, Rho-like GTPases, and protein phosphatase 2 A) [12]. Notably, under Th9-skewing conditions, p38 MAPKs are phosphorylated, which is necessary for *Il9* transcription, and the phosphorylation of p38 MAPK prevents Th9 differentiation *via* FAS signaling [13, 14].

Upon activation, the metabolic requirements of T cells dramatically increase to support the biosynthesis of intracellular constituents, including lipid membranes, nucleic acids, and proteins [15, 16]. Therefore, activated T cells reprogram their metabolic pathways to meet the bioenergetic demand for rapid proliferation [15–17]. For example, lipid metabolism and mammalian target of rapamycin (mTOR) signaling work together to control the fate determination of T-cell subsets [18–20]. mTOR signaling is regulated by various pathways, including the TGF-β signaling and T-cell receptor (TCR) pathways, *via* activation of the PI3K/Akt pathway [21, 22]. In addition, the PI3K/Akt and TGF-β–SMAD pathways act synergistically to control sterol regulatory element-binding protein 1 (SREBP1) activation [23, 24]. SREBP1 is an essential transcriptional regulator of lipid biosynthesis that induces the transcription of genes involved in fatty acid and cholesterol synthesis, such as acetyl-CoA carboxylase 1 (ACC1, the product of the *Acaca* gene), ATP-citrate synthase, fatty acid synthase, and low-density lipoprotein receptor [25]. Thus, lipid metabolism in T cells is intricately regulated by the mTOR and TGF-β signaling pathways.

Although increasing evidence has highlighted the importance of lipid metabolism for T-cell differentiation and function [26–29], the role of lipid metabolism in Th9 cell differentiation remains largely unclear. To address this gap in the literature, we examined the role of lipid metabolism in Th9 cell differentiation and function in vitro in mouse and human cells and in vivo in murine models of melanoma and adenocarcinoma. We found that genetic deletion of T-cell–intrinsic ACC1 or pharmacological inhibition of endogenous ACC1 increased IL-9 production both in mice and humans. In addition, we found that a lack of de novo fatty acid biosynthesis augmented the antitumor immunity of Th9 cells *via* an increase in IL-9 production in mouse tumor models. Mechanistic studies revealed that the increase in IL-9 was strongly dependent on TGF-β signaling. Indeed, under the condition of hindered de novo fatty acid synthesis by targeting ACC1, TGF-β alone was sufficient to induce high levels of IL-9 production by T cells. We also found that de novo fatty acid synthesis regulated *Il9* transcription through control of the retinoic acid signaling pathway. Finally, we found that upon adoptive transfer, ACC1-inhibited Th9 cells suppressed tumor growth in murine models of melanoma and adenocarcinoma. In summary, our findings highlight a novel role of ACC1 in controlling Th9 cell differentiation, which may have clinical implications in cancer therapy.

## Results

### Fatty acid metabolism controls Th9 cell differentiation in vitro

First, we confirmed that fatty acid metabolism is involved in the differentiation of murine Th9 cells by examining the expression of four genes (*Ldlr*, *Lrp8*, *Scarb1*, and *Vldlr*) that encode enzymes involved in fatty acid uptake via quantitative real-time PCR (qRT‒PCR) analysis. Compared with that in naïve CD4 + T cells, the expression of all four genes was significantly upregulated in Th0 (Th cells generated under neutral conditions) and Th9 cells, indicating that fatty acid metabolism is indeed involved in the differentiation of murine Th9 cells (Supplementary Fig. 1a).

**Fig. 1:**
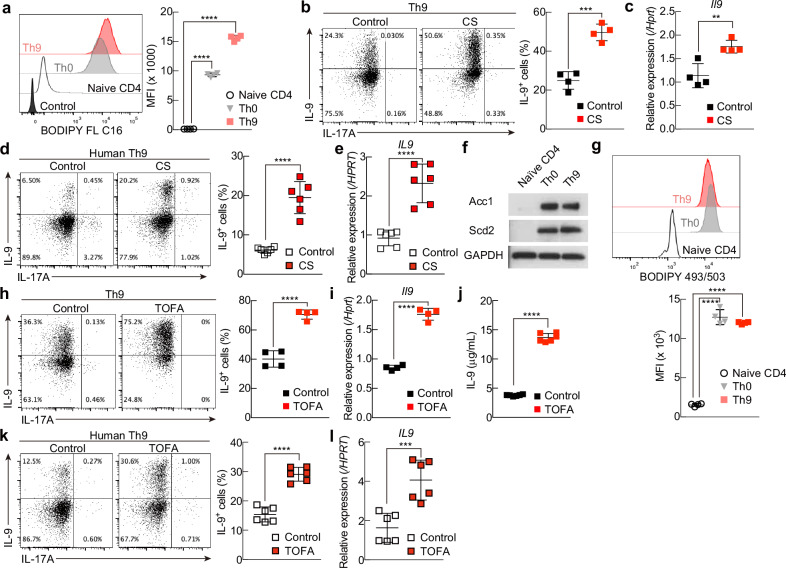
Depletion of environmental fatty acids and inhibition of de novo fatty acid biosynthesis increase IL-9 production by Th9 cells. **A** Representative plots of BODIPY FLC16 in CD4^+^ T cells, including naïve CD4^+^, Th0 and Th9 cells. **B** Representative intracellular staining profiles of IL-9 and IL-17A in Th9 cells cultured in serum from patients with CS. **C** Quantitative RT‒PCR analysis of *Il9* in Th9 cells cultured as described in (**B**). **D** Representative intracellular staining profiles of IL-9 and IL-17A in human Th9 cells cultured in serum from patients with CS. **E** Quantitative RT‒PCR analysis of *IL9* in human Th9 cells cultured as described in (**D**). **F** Western blot analysis of Acc1 and Scd2 in CD4^+^ T cells. **G** Representative plots of BODIPY 493/503 in CD4^+^ T cells, including naïve CD4^+^, Th0 and Th9 cells. **H** Representative intracellular staining profiles of IL-9 and IL-17A in Th9 cells treated with or without TOFA. **I** Quantitative RT‒PCR analysis of *Il9* in Th9 cells cultured as described in (**H**). **J** IL-9 levels in the supernatants on day 3 were tested via ELISA. **K** Representative intracellular staining profiles of IL-9 and IL-17A in human Th9 cells treated with or without TOFA. **l** Quantitative RT‒PCR analysis of *IL9* in human Th9 cells cultured as described in (**K**). *n* = 4 (**A**–**C**), (**G**–**I**); 6 (**D**, **E**), (**J**–**L**) for each group, biologically independent samples are shown. More than three independent experiments were performed with similar results for (**A**–**L**). Mean values with s.d. are shown for (**A**–**E**) and (**G**–**I**). An unpaired two-tailed Student’s t-test was applied for (**A**–**E**) and (**G**–**l**). Statistical significance (*P*-value) is indicated as *P < 0.05; **P < 0.01; ***P < 0.001; ****P < 0.0001; N.S Not significant

Next, we confirmed that murine Th cells acquire free fatty acids from the external environment by examining the uptake of fluorescently labeled palmitate (BODIPY FL C16; Invitrogen). Consistent with the results of the qRT‒PCR analysis, Th0 and Th9 cells acquired significantly higher levels of palmitate from the external environment than did naïve CD4^+^ T cells (Fig. 1A), indicating that the fatty acid uptake program is activated during the differentiation of Th0 and Th9 cells.

We then examined IL-9 production by murine Th9 cells and found that the proportion of IL-9-producing cells was significantly greater under charcoal-stripping (CS) conditions, which reduce the amount of lipids in serum, than under control conditions (Fig. 1B). Consistent with this finding, the expression of *Il9* mRNA was also significantly greater in murine Th9 cells cultured in serum-free medium (CS) than in control Th9 cells (Fig. 1C). Similar results were also found in human Th9 cells (Fig. 1D, E). We also examined the mRNA expression of several transcription factors known to be involved in the differentiation of mouse and human Th9 cells and found that the expression of *Batf3*/*BATF3* was significantly increased in both types of Th9 cells cultured under CS serum conditions (Supplementary Fig. 1b, c).

We next examined the involvement of de novo fatty acid biosynthesis in the differentiation of murine Th9 cells. Compared with those in naïve CD4 + T cells, qRT‒PCR analysis revealed significant upregulation of several mRNAs encoding enzymes involved in fatty acid biosynthesis, including *Acaca*, *Scd1*, *Scd2*, *Acsl3*, *Elovl1*, *Elovl5*, and *Fads2*, in Th9 cells (Supplementary Fig. 1d). We also observed increased levels of ACC1 and SCD2 in murine Th0 and Th9 cells (Fig. 1F), as well as a large, significant increase in lipid droplets in these cells compared with those in naïve CD4^+^ T cells (Fig. 1G and Supplementary Fig. 1e).

To confirm that fatty acid biosynthesis plays a role in the differentiation and function of Th9 cells, we treated mouse and human Th9 cells with 5-(tetradecyloxy)-2-furoic acid (TOFA), a pharmacological inhibitor of ACC1. In both mouse and human Th9 cells, IL-9 production and *Il9* mRNA expression were significantly greater in the TOFA-treated group than in the untreated control group (mouse, Fig. 1H–J; human, Fig. 1K, L). Furthermore, TOFA treatment significantly increased the mRNA expression of several transcription factors associated with the differentiation of Th9 cells: mouse *Batf3* and *Irf4* and human *BATF3*, *IRF4*, and *SPI1* (Supplementary Fig. 1f, g).

Together, these results indicate that the fatty acid uptake program and de novo fatty acid biosynthesis are both deeply involved in the differentiation and function of mouse and human Th9 cells.

### Extrinsic supplementation with fatty acids restores IL-9 production in *Acaca*^ΔT^ Th9 cells

**Fig. 2:**
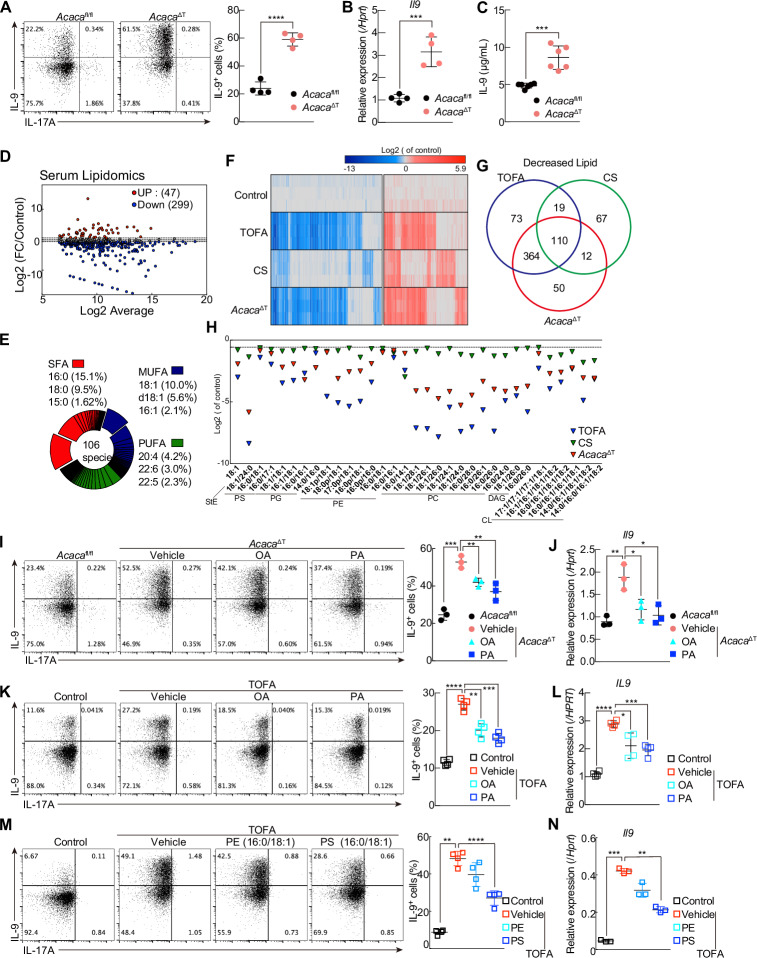
Increased IL-9 production in de novo fatty acid biosynthesis-depleted Th9 cells is restored by supplementation with monounsaturated or saturated fatty acids. **A** Representative intracellular staining profiles of IL-9 and IL-17A in Th9 cells. The cells were collected from *Acaca*^fl/fl^ or *Acaca*^ΔT^ mice and cultured under Th9 conditions for 3 days in vitro. **B** Quantitative RT‒PCR analysis of *Il9* in Th9 cells cultured as described in (**A**). **C** IL-9 levels in the supernatants on day 3 were tested via ELISA. **D** Dot plot depicting the ratio of changed lipids in CS serum to the control. **E** Pie chart showing the percentage of a total of 106 species of FAs incorporated into lipids, which were reduced in CS serum, as depicted in (**D**). Data are partitioned on the basis of the length and saturation of their fatty acyl side chains. Those carrying more than one fatty acid are further grouped according to least saturation or the longest carbon chain. SFA, saturated fatty acid; MUFA, monounsaturated fatty acid; PUFA, polyunsaturated fatty acid. **F** Heatmap depicting the altered lipid composition in control, *Acaca*^ΔT-treated^ and TOFA-treated Th9 cells. Three biological replicates of each group are shown. **G** Venn diagram depicting the number of decreased lipids in CS-treated, TOFA-treated and *Acaca*^ΔT^ Th9 cells compared with control cells. **H** The proportions of lipids that satisfy the following criteria are shown. Compared with those in control cells, lipids in CS-serum, TOFA-treated and *Acaca*^ΔT^ Th9 cells are commonly decreased, as shown in (**G**). Among the commonly decreased lipids, the lipids included specific fatty acids that were present in more than 10% of the lipids that were reduced in the CS serum, as shown in (**E**). **I** Representative intracellular staining profiles of IL-9 and IL-17A in Th9 cells treated with or without TOFA plus OA (50 mM) or PA (25 mM). **J** Quantitative RT‒PCR analysis of *Il9* in Th9 cells cultured as described in (**I**). **K** Representative intracellular staining profiles of IL-9 and IL-17A in human Th9 cells treated with or without TOFA plus OA (50 mM) or PA (25 mM). **l** Quantitative RT‒PCR analysis of *IL9* in human Th9 cells cultured as described in (**K**). **M** Representative intracellular staining profiles of IL-9 and IL-17A in Th9 cells treated with or without TOFA plus [PE (16:0/18:1)] (30 mM) or [PS (16:0/18:1)] (30 mM) in the presence of methyl beta cyclo dextrin (0.01 mM). **N** Quantitative RT‒PCR analysis of *Il9* in Th9 cells cultured as described in (**M**); *n* = 3 (**F**, **G** **H**); 4 (**A**), (**B**), (**D**), (**I**–**N**); and 6 (**C**) biologically independent samples from each group are shown. More than two independent experiments were performed with similar results for (**A**–**C**) and (**I**–**N**). Mean values with s.d. are shown for (**A**–**C**) and (**I**–**N**). An unpaired two-tailed Student’s t-test was applied for (**A**–**C**) and (**I**–**N**). Statistical significance (*P*-value) is indicated as *P < 0.05; **P < 0.01; ***P < 0.001; ****P < 0.0001; N.S. not significant

To directly assess the role of ACC1-mediated fatty acid biosynthesis in Th9 cell differentiation, we used mice in which the biotin carboxyl carrier protein domain in the *Acaca* gene in CD4^+^ T cells was conditionally deleted by the expression of Cre recombinase driven by the *Cd4* promoter (hereafter referred to as *Acaca*^ΔT^) [26, 30]. We observed a statistically significant 2- to 3-fold increase in the proportion of IL-9–producing cells and a significant increase in the expression of *Il9* in murine *Acaca*^ΔT^ Th9 cells compared with that in murine *Acaca*^fl/fl^ Th9 cells (Fig. 2A–C). *Batf3* expression was also significantly increased in murine *Acaca*^ΔT^ Th9 cells compared with *Acaca*^fl/fl^ Th9 cells (Supplementary Fig. 2a).

We next investigated which fatty acids are directly involved in the differentiation of murine Th9 cells. Since defects in FA biosynthesis or a reduction in extracellular lipids augment IL-9 production, we performed lipidomic analysis to reveal the lipidome profile of cultured serum and differentiated Th9 cells. Serum lipidomics identified 422 lipid species, of which 299 were decreased in CS serum (Fig. 2D). Furthermore, examining the fatty acids associated with decreased lipids, we found that palmitic acid [PA (16:0), 15.1%)] and oleic acid [OA (18:1), 10.0%] were predominantly detected in high amounts (criterion: > 10.0% of total fatty acids) (Fig. 2E). A global lipidomic analysis revealed a total of 1165 lipid species in Th9 cells (Fig. 2F). We also found that CS serum, TOFA-treated and *Acaca*^ΔT^ Th9 cells increased 253, 295, and 344 and reduced 208, 566 and 536 lipid species, respectively (Fig. 2F, G; Supplementary Fig. 2b; and Supplementary Table 1). A comparison revealed that 110 lipids were commonly decreased in CS-treated, TOFA-treated and *Acaca*^ΔT^ Th9 cells (Fig. 2G). Since serum lipidomics suggests the importance of PA and OA in IL-9 production, we focused on whether the modulation of lipid metabolism in Th9 cells decreases the amount of lipids that contain these fatty acids. Consequently, 33 of 110 lipids were selected as candidates for the regulation of IL-9 production. Most lipids are phosphatidylcholine (PC), phosphatidylserine (PS), phosphatidylglycerol (PG), cardiolipin (CL), and diacylglycerol (DAG). Among the commonly reduced lipids, only PS, PE and CL contained both PA and OA (Fig. 2H).

Previously, we reported that extrinsic supplementation with fatty acids restored the function of *Acaca*^ΔT^ Th17 cells and improved the proliferation, survival, and metabolic reprogramming of TOFA-treated activated CD4^+^ T cells [17, 26, 31]. To confirm the lipidomic data, we next sought to determine whether supplementation with fatty acids would prevent the differentiation induced in Th9 cells cultured under fatty acid–limited conditions. As expected, in *Acaca*^ΔT^ murine Th9 cells, supplementation with OA or PA, the most depleted component, partially suppressed the increase in the proportion of IL-9-producing cells and the increase in *Il9* expression (Fig. 2L, J), whereas extrinsic supplementation of stearic acid [SA (18:0)] into TOFA-treated Th9 cell cultures failed to suppress IL-9 production (Supplementary Fig. 2c). We also investigated the effects of seven fatty acids, including arachidonic acid (20:4), which is the most abundant PUFA (accounting for as much as 4.2% of total fatty acids), and docosahexaenoic acid, which is a typical polyunsaturated fatty acid. However, none of these compounds inhibited IL-9 production by TOFA-treated Th9 cells (Supplementary Fig. 2d, e). Similar results were found in TOFA-treated human Th9 cells (Fig. 2K, L). Finally, we investigated the types of lipids that are crucial for the regulation of IL-9 production. Importantly, the administration of PS [16:0/18:1], but not PE [16:0/18:1], suppressed the production of IL-9 in TOFA-treated Th9 cells (Fig. 2M, N). Although there was a substantial reduction in the amount of CL containing OA or PA in CS-treated, TOFA-treated, and *Acaca*^ΔT^ Th9 cells, the inhibition of CL synthesis did not increase IL-9 production (Supplementary Fig. 2f). Taken together, a decrease in lipids containing PA or OA, such as PS [16:0/18:1], contributes to the increase in IL-9 production. Thus, supplementation with a saturated or monounsaturated fatty acid prevented the increase in IL-9 production induced by the pharmacological inhibition or genetic deletion of ACC1 in mouse and human Th9 cells.

### ACC1 controls the permissive chromatin landscape at the *Il9* gene locus

Since Th17-cell skewing conditions (IL-6 plus TGF-β) are somewhat similar to those for Th9 cells (IL-4 plus TGF-β), we differentiated naïve CD4^+^ T cells into Th17 cells in the presence of TOFA in vitro and observed that Th17 cells significantly produced much more IL-9 when *Acaca* was pharmacologically inhibited (Supplementary Fig. 3a).

**Fig. 3:**
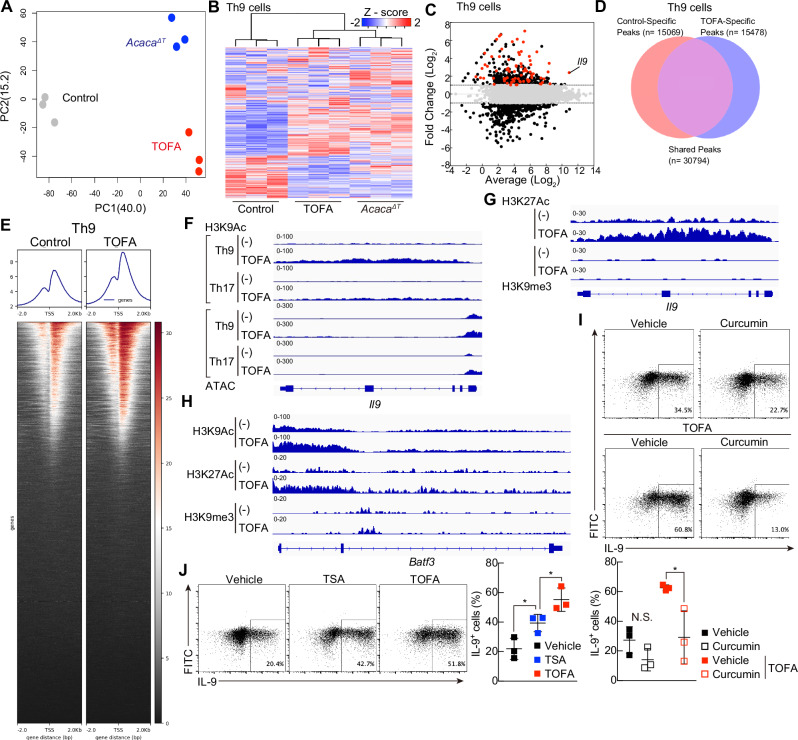
ACC1 represses the permissive chromatin landscape at the *Il9* gene locus (**A**) PCA plot of gene expression profiles obtained via RNA sequencing in control, TOFA-treated or *Acaca*^ΔT^ Th9 cells. **B** A clustering heatmap depicting the genes in control, TOFA-treated or *Acaca*^ΔT^ Th9 cells. **C** MA plot analysis of RNA-seq data from control and TOFA-treated Th9 cells. The red dots indicate genes associated with retinoic acid-related genes. The set of genes whose expression increased more than 2-fold in RARα KO Th9 cells was defined from a previously published dataset (GSE123501). **D** Venn diagram showing overlaps and differences in peaks between control and TOFA-treated Th9 cells via ChIP-seq. **E** Average plots and heatmaps showing H3K9ac enrichment at the TSS in the ChIP-seq datasets. **F**–**H** Representative *Il9* or *Batf3* gene tracks via RNA-seq and ChIP-seq (H3K9ac, H3K27ac, or H3K9me3) in Th9 or Th17 cells polarized in the presence of the vehicle control or TOFA. **I** Representative intracellular staining profiles of IL-9 in Th9 or TOFA-Th9 cells treated with or without curcumin (2.5 mM). **J** Representative intracellular staining profiles of IL-9 in Th9 cells treated with vehicle control, TSA (3 nM) or TOFA. *n* = 1 (**D**, **E**) and (**F**–**H**); 3 (**A**–**C**), (**I**, **J**) for each group, biologically independent samples are shown. Two biologically independent experiments were performed with (**D**–**H**). More than three independent experiments were performed with similar results for (**I**, **J**). The mean values with s.d. are shown for (**I**, **J**). An unpaired two-tailed Student’s t-test was applied for (**I**, **J**). Statistical significance (*P*-value) is indicated as *P < 0.05; **P < 0.01; ***P < 0.001; ****P < 0.0001; N.S. not significant

To better understand the effects of fatty acid metabolism on the Th9 cell program, we performed RNA-seq analysis of Th9 and Th17 cells differentiated under control, TOFA-treated, or *Acaca*^ΔT^ conditions. Principal component analysis revealed that TOFA-treated or *Acaca*^ΔT^ Th9 or Th17 cells formed clusters that were distinct from those of control Th9 cells (Fig. 3A and Supplementary Fig. 3b). A heatmap obtained via unsupervised hierarchical clustering revealed many upregulated and downregulated genes in the cells cultured under TOFA-treated or *Acaca*^ΔT^ conditions compared with the control cells, suggesting extensive Th9 reprogramming under these conditions (Fig. 3B). We identified 1056/865 differentially expressed genes (DEGs) among control, TOFA, and *Acaca*^ΔT^ Th9 cells, including 495/449 genes whose expression was upregulated and 479/341 genes whose expression was downregulated (Fig. 3B). A similar tendency was observed for TOFA-treated and *Acaca*^ΔT^ Th17 cells (Supplementary Fig. 3c). MA plots revealed that *Il9* was one of the genes whose expression was most highly upregulated in TOFA-treated Th9 and Th17 cells (Fig. 3C and Supplementary Fig. 3d).

Murine Th9 cell differentiation is known to be controlled by several Th9-associated transcription factors, including *Spi1*, *Irf4*, *Batf*, *Batf3, Stat5*, *Stat6*, *Hif1α*, and *Foxo1* [32–34]. Compared with those in control cells, the expression of two of these factors, *Irf4* and *Batf3*, was greater in TOFA-treated cells than in control cells according to RNA-seq analysis, similar to the results of earlier qRT‒PCR analysis (Supplementary Fig. 1d), but the expression of the other factors remained largely unchanged (Supplementary Fig. 3e). In response to these results, we examined whether CRISPR-mediated deletion of *Batf3* in TOFA-treated murine Th9 cells prevents the increase in IL-9 production; however, no changes in IL-9 production were detected (Supplementary Fig. 3f).

Epigenetic chromatin modifications can selectively control the expression of genes that function in the immune system [35]. We therefore explored whether ACC1-mediated fatty acid biosynthesis regulates the chromatin status of Th9-associated gene loci in Th9 cells. We performed chromatin immunoprecipitation sequencing (ChIP-seq) analysis of H3K9 acetylation (ac) to evaluate the changes in the epigenetic landscape in detail. Examination of the profile revealed more than 10,000 unique peaks in control and TOFA-treated Th9 and Th17 cells, and the H3K9ac profiles around the transcription start sites of the genes showed marked differences (Fig. 3D, E and Supplementary Fig. 3g–i). We also used ATAC-seq (assay for transposase-accessible chromatin by sequencing) to examine the permissive chromatin landscape in control and TOFA-treated Th9 and Th17 cells. The chromatin in the *Il9* promoter region was in a permissive state in TOFA-treated Th9 and Th17 cells (Fig. 3F). Genome-wide H3K9ac and ATAC-seq signal profiles revealed striking increases in the levels and extent of epigenetic marks at the *Il9* gene locus in TOFA-treated Th9 and Th17 cells (Fig. 3F). Furthermore, TOFA-treated Th9 cells presented exceptionally high levels of H3K9ac, which spans a large genomic segment (Fig. 3F). To explore whether pharmacological inhibition of ACC1 affects the epigenetic chromatin status as well as H3K9ac, we subsequently conducted ChIP-seq analysis using anti-H3K27ac or anti-H3K9 trimethylation (H3K9me3) antibodies. Although western blotting analysis revealed no significant difference in overall H3K27ac and H3K9me3 levels between control and TOFA-treated Th9 cells, TOFA treatment increased H3K27Ac levels at the *Il9* gene locus (Fig. 3G and Supplementary Fig. 3j–m). In addition to *Il9*, the suppression of fatty acid biosynthesis increased the levels of H3K9ac and H3K27Ac at the *Batf3* gene locus (Fig. 3H). We barely detected H3K9me3 signals at the *Il9* and *Batf3* gene loci in both control and TOFA-treated Th9 cells, indicating that these gene loci are transcriptionally active in differentiated Th9 cells (Fig. 3G, H). To evaluate the importance of histone acetylation for IL-9 production, we examined whether the administration of the potent histone acetyltransferase inhibitor curcumin suppressed IL-9 production in TOFA-treated Th9 cells and found that curcumin significantly suppressed it (Fig. 3I). In addition, treatment with trichostatin A, a representative histone deacetylase inhibitor, significantly increased IL-9 production compared with that of the control, but the increase was not as high as that induced by TOFA treatment (Fig. 3J).

Together, these results indicate that the inhibition of ACC1-mediated de novo fatty acid biosynthesis results in a permissive chromatin landscape at the *Il9* gene locus that is critical for robust Th9 cell differentiation.

### ACC1-mediated fatty acid biosynthesis controls the TGF-β–Smad2/3 pathway and limits Th9 cell differentiation

**Fig. 4:**
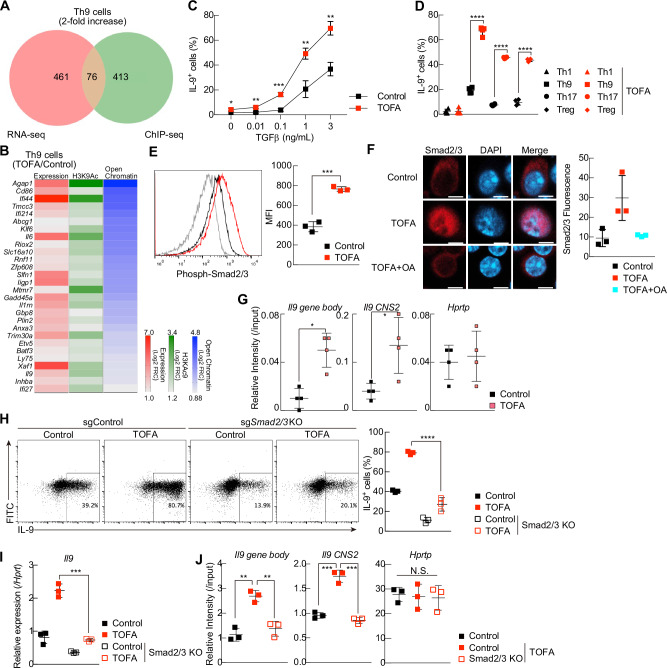
Increased IL-9 production in TOFA-Th9 cells is dependent on the TGF-β–Smad2/3 pathway. **A** Venn diagram showing overlaps and differences between 2.0-fold increased genes (RNA-seq or ChIP-seq; H3K9ac) in TOFA-treated Th9 cells relative to control Th9 cells. **B** Commonly upregulated genes identified via RNA-seq and ChIP-seq, related to (**A**), are shown here. Left columns, expression; middle columns, histone acetylation; right columns, permissive chromatin landscape in the TOFA/WT ratio. **C** The graph shows IL-9 production in Th9 cells cultured with the indicated concentrations of TGF-β, as analyzed via FACS. **D** The graph shows IL-9 production in Th cell subsets cultured with or without TOFA as analyzed by FACS. **E** Intracellular staining of phospho-Smad2/3 in Th9 or TOFA-Th9 cells. **F** Immunofluorescence analyses were performed with anti-Smad2/3 antibodies and DAPI in TOFA-Th9 cells in the presence or absence of OA (50 mM) and in control Th9 cells. The scale bar represents 5 mm. The fluorescence intensity of anti-Smad2/3 in the nucleus was calculated via ImageJ software. **G** ChIP assays were performed with an anti-Smad2 antibody at the *Il9* locus in Th9 cells. The intensities of these modifications relative to input DNA were determined via quantitative RT‒PCR analysis. **H** Representative intracellular staining profiles of IL-9 in control and TOFA Th9 cells treated with sgControl or sgSmad2/3. **I** Quantitative RT‒PCR analysis of *Il9* in Th9 cells cultured as described in (**H**). **J** ChIP assays were performed with an anti-H3K9ac antibody at the *Il9* locus in Th9 cells. The intensities of these modifications relative to input DNA were determined via quantitative RT‒PCR analysis. *n* = 3 (**E**, **F**, **H**, **I**, **J**); (**C**, **D**, **G**) for each group, biologically independent samples are shown. Two biologically independent experiments were performed with (**A**, **B**, **J**). More than three independent experiments were performed with similar results for (**C**–**J**). The mean values with s.d. are shown for (**C**, **D**), and (**E**–**J**). An unpaired two-tailed Student’s t test was applied for (**C**–**E**) and (**G**–**J**). Statistical significance (*P* value) is indicated as *P < 0.05; **P < 0.01; ***P < 0.001; ****P < 0.0001; N.S. not significant

To understand more about the mechanisms underlying ACC1-mediated fatty acid biosynthesis, we used a combination of genome-wide RNA-seq and ChIP-seq profiling. Thus, we first checked the overlap of the peaks between the control and TOFA-treated Th9 cells and obtained 15478 (*n* = 1) or 17105 (*n* = 2) increased peaks and 15069 (*n* = 1) or 12124 (*n* = 2) decreased peaks (Fig. 3D and Supplementary Fig. 3g). Among the 15478 (*n* = 1) or 17105 (*n* = 2) TOFA-specific peaks, 1915 (*n* = 1) or 2019 (*n* = 2) were identified as differentially increased peaks (criteria: fold change > 2.0, *P*-value < 0.0001). We also confirmed that these differential peaks included 1576 (*n* = 1) or 1715 (*n* = 2) increased genes. Finally, to confirm reproducibility, we extracted 489 commonly increased genes in two independent experiments. Similarly, we also found that 188 genes presented decreased H3KAc9 levels (Supplementary Fig. 4a). A Venn diagram of the combination of RNA-seq and ChIP-seq analyses revealed that the expression of 76 and 109 genes, including *Il9*, was increased in TOFA-treated murine Th9 and Th17 cells compared with control cells (Fig. 4A, B; Supplementary Fig. 4a–c; and Supplementary Table 2). Among the 76 genes whose expression changed in Th9 cells, 42 genes were shared with those identified in Th17 cells, nearly half of which were related to the TGF-β signaling pathway and included *Il9*, *Gadd45a*, *Il1rn*, and *Tmcc3* [36–39] (Fig. 4B, Supplementary Fig. 4c, and Supplementary Table 2).

To examine how ACC1-mediated fatty acid biosynthesis controls murine Th9 cell differentiation, we investigated the differentiation of TOFA-treated murine Th9 cells cultured with different concentrations of IL-4 and TGF-β. TOFA-treated Th9 cells were able to produce IL-9 even in the absence of IL-4 (Supplementary Fig. 4d). In addition, the IL-4 concentration had only a mild effect on IL-9 production, especially in TOFA-treated Th9 cells (Supplementary Fig. 4e). Unlike the limited effect of IL-4 on Th9 cell differentiation, TGF-β significantly increased IL-9 production in a dose-dependent manner, especially in TOFA-treated Th9 cells (Fig. 4C and Supplementary Fig. 4F). Th17 and regulatory T cells, which are both reliant upon TGF-β for their differentiation, exhibited a significant increase in IL-9 production following treatment with TOFA, whereas no such effect was detected in Th1 cells (Fig. 4D and Supplementary Fig. 4g).

The TGF-β–Smad2/3 signaling pathway regulates several transcription factors that participate in the differentiation of Th9 cells, including PU.1 and Batf3 [7]. We therefore next assessed the involvement of Smad2/3 in TOFA-treated murine Th9 cells. The phosphorylation of Smad2/3 in these cells was significantly greater than that in control Th9 cells (Fig. 4E). Immunofluorescence microscopy revealed that the nuclear localization of Smad2/3 was increased by treatment with TOFA and that this effect was reversed by oleic acid supplementation in TOFA-treated murine Th9 cells (Fig. 4F). Consistent with these observations, TOFA treatment increased the binding of Smad2 to the regulatory element of the *Il9* gene locus (Fig. 4G). Furthermore, CRISPR/Cas9-mediated double knockout of Smad2/3 in TOFA-treated murine Th9 cells resulted in a significant reduction in IL-9 production (Fig. 4H, I). We also found that double knockout of Smad2/3 prevented TOFA-mediated H3K9ac at the *Il9* gene locus (Fig. 4J).

The TAK1–ID3 pathway, which is a SMAD-independent TGF-β signaling pathway, also contributes to the differentiation of Th9 cells [10, 11]. Thus, we investigated whether this pathway could be involved in the Th9 cell differentiation induced by the inhibition of ACC1. Consistent with previous reports, a TAK1 inhibitor (5Z-7-oxozeaenol; MCE) significantly decreased IL-9 production in control Th9 cells but had a minimal effect on IL-9 production in TOFA-treated cells (Supplementary Fig. 4h).

### ACC1-mediated fatty acid biosynthesis inhibits Th9 cell differentiation *via* retinoic acid–retinoic acid receptor-alpha signaling

**Fig. 5:**
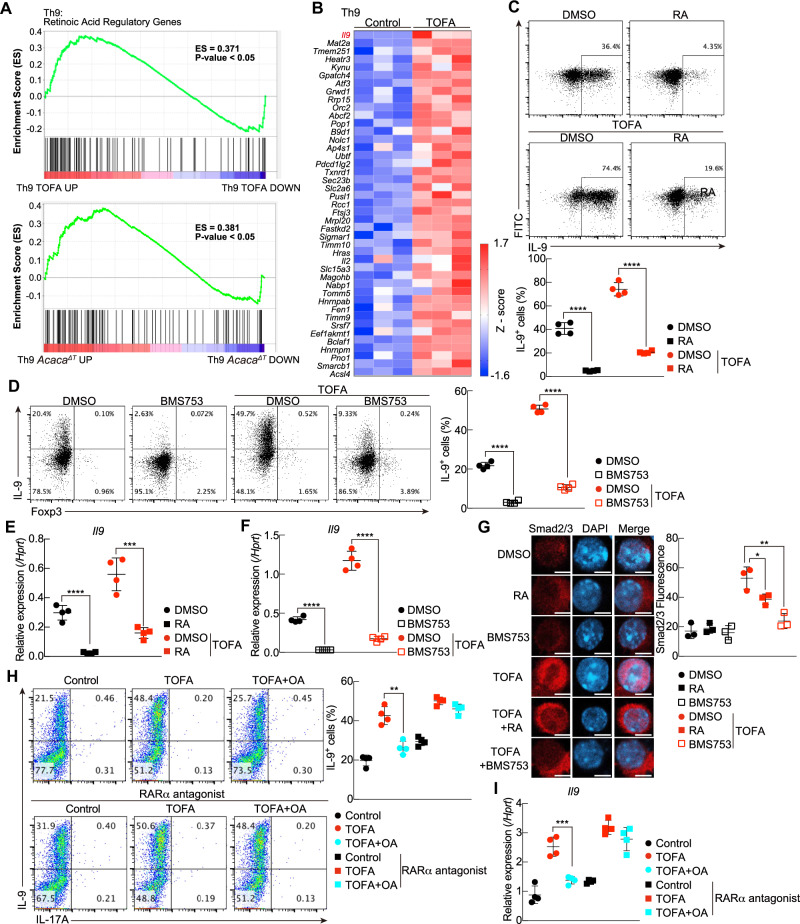
A lack of ACC1-mediated fatty acid biosynthesis increases IL-9 production *via* the inhibition of RA-RARα signaling. **A** Gene set enrichment analysis (GSEA) revealed decreased expression of genes encoding retinoic acid-related genes in Th9 cells upon treatment with TOFA (top) or *Acaca*^ΔT^ Th9 cells (bottom). **B** Heatmap depicting genes differentially expressed in the retinoic acid regulatory genes in control and TOFA-treated Th9 cells. **C** Representative intracellular staining profiles of IL-9 in control and TOFA-treated Th9 cells with or without RA (1 mM). **D** Representative intracellular staining profiles of IL-9 in control and TOFA Th9 cells with or without BMS753. **E** Quantitative RT‒PCR analysis of *Il9* in Th9 cells cultured as described in (**C**). **F** Quantitative RT‒PCR analysis of *Il9* in Th9 cells cultured as described in (**E**). **G** Immunofluorescence analyses were performed with anti-Smad2/3 antibodies and DAPI in TOFA-Th9 cells in the presence or absence of RA (1 mM) or BMS753 and in control Th9 cells. The scale bars represent 5 mm. The fluorescence intensity of anti-Smad2/3 in the nucleus was calculated via ImageJ software. **H** Representative intracellular staining profiles of IL-9 and IL-17A in control, TOFA and TOFA plus OA Th9 cells with or without the RARα antagonist. **I** Quantitative RT‒PCR analysis of *Il9* in Th9 cells cultured as described in (**H**). *n* = 3 (**A**), (**B**); 4 (**C**–**F**), (**H**, **I**) for each group, biologically independent samples are shown. More than three independent experiments were performed with similar results for (**A**–**I**). The mean values with s.d. are shown for (**C**–**I**). An unpaired two-tailed Student’s t-test was applied for (**C**–**F**) and (**H**, **I**). Statistical significance (*P*-value) is indicated as *P < 0.05; **P < 0.01; ***P < 0.001; ****P < 0.0001; N.S. not significant

To gain more insight into the effects of the inhibition of fatty acid biosynthesis in Th9 cells, we evaluated the DEGs in TOFA-treated or *Acaca*^ΔT^ Th9 and Th17 cells. Gene set enrichment analysis revealed statistically significant enrichment of retinoic acid regulatory genes in TOFA-treated and *Acaca*^ΔT^ Th9 cells (Fig. 5A and Supplementary Fig. 5a). In particular, the expression of a group of genes whose expression is suppressed in the presence of retinoic acid (RA) in Th9 cells was found to be upregulated by TOFA treatment (Fig. 5B and Supplementary Fig. 5b) [33].

Because RA is known to act through its receptor, RARα, to inhibit the Th9 transcriptional program [33], we hypothesized that RA–RARα signaling is involved in ACC1-mediated suppression of Th9 cell differentiation. RA administration significantly decreased IL-9 production in TOFA-treated Th9 cells (Fig. 5C). Similarly, treatment of TOFA-treated Th9 cells with BMS753, a selective agonist of RARα [33], significantly suppressed Th9 cell differentiation (Fig. 5D). Furthermore, *Il9* expression was also significantly reduced by the administration of RA or BMS753 to TOFA-treated Th9 cells (Fig. 5E, F). Consistent with these results, immunofluorescence microscopy revealed that the nuclear localization of Smad2/3 in TOFA-treated Th9 cells was prevented by treatment with RA or BMS753 (Fig. 5G). We found that the phosphorylation of Smad2/3 induced by TOFA was partially suppressed by RA treatment (Supplementary Fig. 5c, d).

Conversely, BMS195614, a selective RARα antagonist, has been shown to increase IL-9 production [33]. Therefore, to further evaluate the effect of RA–RARα signaling on fatty acid metabolism, BMS195614 was added to TOFA-treated Th9 cell cultures supplemented with oleic acid. Oleic acid abrogated IL-9 production in TOFA-treated Th9 cells but not in the presence of BMS195614 (Fig. 5H, I). Although RA treatment increased RARα binding to the *Il9* gene locus, as reported previously [33], TOFA treatment disrupted RARα binding to its target locus (Supplementary Fig. 5e).

Together, these data indicate that ACC1-mediated fatty acid biosynthesis inhibits IL-9 production by reducing the nuclear localization of Smad2/3 and inhibiting RARα binding to the *Il9* gene locus via ΡΑ–ΡΑΡα signaling.

### Pharmacologic inhibition of ACC1 enhances Th9 cell differentiation and facilitates IL-9–dependent antitumor activity in vivo

**Fig. 6:**
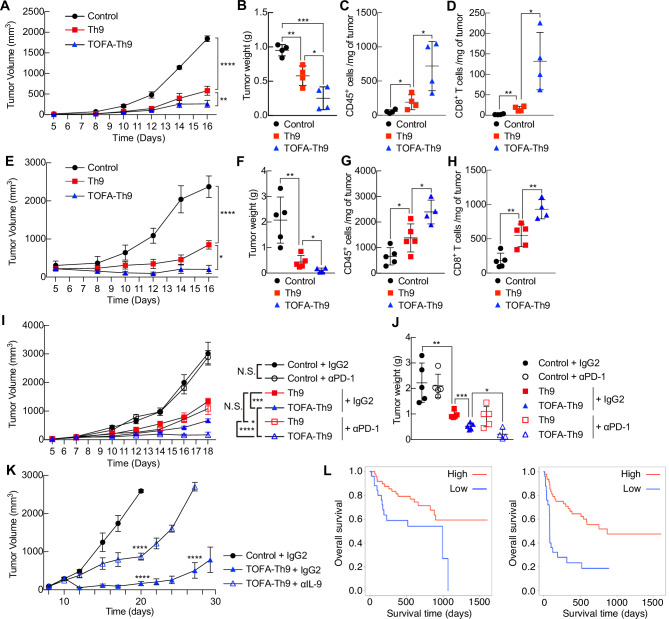
Compared with control Th9 cells, tumor-specific TOFA-Th9 or combination therapy with aPD-1 and TOFA-Th9 cells efficiently induced tumor rejection. **A** Tumor responses to OT-II Th9 cell transfer in the B16 tumor model are shown. **B** Tumor weights at the end of the experiment from (**A**). **C** The number of CD45^+^ cells per mg of tumor in (**A**). **D** The number of CD8^+^ T cells per mg of tumor in (**A**). **E** Tumor responses to OT-II Th9 cell transfer in the MC38 tumor model are shown. **F** Tumor weights at the end of the experiment from (**E**). **G** The number of CD45^+^ cells per mg of tumor in (**E**). **H** The number of CD8^+^ T cells per mg of tumor in (**E**). **I** Tumor responses to combination therapy consisting of OT-II Th9 cell transfer and an anti-PD-1 antibody in the B16 tumor model are shown. **J** Tumor weights at the end of the experiment from (**I**). **K** Tumor responses to OT-II Th9 cell transfer with or without anti-IL-9 antibody treatment in the B16 tumor model are shown. **L** Survival curves according to Th9-related genes are shown. PFS and OS were analyzed via the Kaplan‒Meier method and compared among the groups via the log-rank test. *n* = 4 (**A**–**D**); 5 (**E**–**K**) biologically independent samples from each group are shown. More than three independent experiments were performed with similar results for (**A**–**K**). Mean values with s.d. are shown for (**A**–**K**). Two-way ANOVA was applied for (**A**, **E**, **I**, **K**). An unpaired two-tailed Student’s t test was applied for (**B**–**D**), (**F**–**H**, **J**). Statistical significance (*P*-value) is indicated as *P < 0.05; **P < 0.01; ***P < 0.001; ****P < 0.0001; N.S. Not significant

Having established a function for ACC1-dependent fatty acid biosynthesis in Th9 cell differentiation in vitro, we next determined the roles of ACC1 in Th9 cell–mediated antitumor activity in vivo. Because previous studies have shown that Th9 cells have superior antitumor properties compared with Th1 or Th17 cells upon adoptive cell transfer [6, 40, 41], we first used an experimental mouse model of melanoma in which B6 mice were injected with B16-OVA (ovalbumin (OVA)-transfected B16F10) melanoma cells and subjected to adoptive transfer of OVA-specific OT-II transgenic Th9 cells five days after tumor inoculation (Supplementary Fig. 6a). Consistent with our in vitro experiments, mice that received TOFA-treated cells, which presented increased IL-9 expression, presented significant tumor regression compared with that of mice that received untreated Th9 cells (Fig. 6A, B and Supplementary Fig. 6b). Analysis of the tumor-infiltrated cells revealed that compared with the administration of untreated Th9 cells, the administration of TOFA-Th9 cells resulted in significantly increased infiltration of CD45^+^ cells and CD8^+^ T cells (Fig. 6C, D). We also observed that the numbers of tumor-infiltrating granzyme B- or interferon gamma (IFNγ)-producing CD8^+^ T cells were significantly greater in TOFA-Th9 cells than in untreated Th9 cells (Supplementary Fig. 6c). Furthermore, the number of tumor-infiltrating CD4^+^ T cells was also significantly greater in TOFA-Th9 cells than in untreated Th9 cells (Supplementary Fig. 6d). The tumor-infiltrating CD4^+^ T cells in the TOFA-Th9 group contained more transferred cells than did those in the control Th9 group (Supplementary Fig. 6e). The significantly stronger antitumor activity of TOFA-treated Th9 cells was also observed in an MC38-OVA colon adenocarcinoma tumor model (Fig. 6E, F and Supplementary Fig. 6f, g). Additionally, in agreement with the results obtained with the B16-OVA model, the numbers of tumor-infiltrating CD45^+^, granzyme B- or IFNγ-producing CD8^+^ T cells and CD4^+^ T cells were significantly greater in the TOFA-treated Th9 group than in the untreated Th9 cell group (Fig. 6G, H and Supplementary Fig. 6h, i).

Checkpoint blockade therapy activates antitumor immunity by targeting proteins that inhibit T-cell proliferation and function [42]. We therefore examined combination therapy with TOFA-treated Th9 cell transfer and checkpoint antibody blockade. We transplanted B16-OVA cells into mice that received Th9 cells with or without TOFA treatment and then treated these mice with isotype or PD-1–blocking antibodies (Supplementary Fig. 6j). Compared with the transfer of TOFA-treated Th9 cells alone, combination therapy with TOFA-treated Th9 cells and PD-1 blockade resulted in significant tumor regression (Fig. 6I, J and Supplementary Fig. 6k, l).

Th9 cells are known to elicit strong host antitumor CD8^+^ cytotoxic T lymphocyte (CTL) responses by promoting Ccl20/Ccr6-dependent recruitment of dendritic cells to tumor tissues *via* IL-9 production [40]. To further investigate the link between Th9 cells and ACC1-mediated fatty acid biosynthesis in the context of cancer, we next treated tumor-inoculated mice with IL-9–neutralizing antibodies (αIL-9). One day before Th9 cell transfer, B6 mice were given one dose of cyclophosphamide to induce temporary lymphopenia, which is frequently induced as part of clinical adoptive cell therapy protocols to promote homeostatic proliferation of transferred T cells (Supplementary Fig. 6m) [6]. Mice also received adjuvant OVA peptide–pulsed dendritic cell vaccination on the day of transfer, which is used to boost the antitumor response during adoptive cell therapy [6]. TOFA-treated Th9 cells mediate tumor regression, resulting in long-term survival (Fig. 6K). Anti-IL-9 antibody treatment significantly abrogated the beneficial effect of TOFA in Th9 cells, indicating that the antitumor activity is likely driven mainly by increased IL-9 production (Fig. 6K and Supplementary Fig. 6n, o).

Finally, we performed publicly available dataset analysis of RNA-seq data derived from 73 patients with melanoma who received anti-PD1 antibodies such as nivolumab or pembrolizumab (Fig. 6L) [43, 44]. We sought to determine whether Th9-related genes, especially those upregulated by ACC1 inhibition, including *IL9*, *BATF3*, *TNFSF8*, *SPI1*, and *IRF4*, were associated with 10-year overall survival (OS) or progression-free survival (PFS). PFS and OS were significantly longer for anti-PD1 antibody-treated patients with high expression of the *IL9*/*BATF3*/*TNFSF8*/*SPI1*/*IRF4* Th9 phenotype than for those with low expression (Fig. 6L).

In summary, the suppression of fatty acid biosynthesis augmented IL-9 production *via* the Smad2/3 and RA-RARα signaling pathways (Supplementary Fig. 7a), which could be a new tool for the clinical study of combination therapy with PD-1 blocking antibodies (Supplementary Fig. 7b).

## Discussion

Here, we report that fatty acid metabolism plays an essential role in murine Th9 cell differentiation and function in vivo. We demonstrated that the restriction of the cellular fatty acid content either by the deprivation of environmental fatty acids or the inhibition of ACC1-mediated fatty acid biosynthesis strongly enhanced murine and human Th9 cell differentiation. Furthermore, the inhibition of fatty acid biosynthesis in murine Th9 cells promoted their antitumor activity in vivo. Interestingly, combination therapy with ACC1-inhibited Th9 cell transfer and PD-1 blockade resulted in superior tumor regression compared with ACC1-inhibited Th9 cell transfer alone. Furthermore, our results showed that blockade of IL-9 signaling significantly abrogated the beneficial effect of ACC1 inhibition in Th9 cells, suggesting that the antitumor activity of Th9 cells could be mediated by increased IL-9 production. Finally, mechanistic analyses revealed that fatty acids inhibited IL-9 production, reducing the nuclear localization of SMAD2/3 and *Il9* transcription *via* RA–RARα signaling.

Lipidomic analysis revealed that defects in FA biosynthesis or incorporation differentially affect the composition of the lipidome profile in Th9 cells. Among the 364 types of decreased lipids in both TOFA-treated and *Acaca*^ΔT^ Th9 cells, 24 species of DAG and 76 species of triacylglycerol (TAG), which are components of lipid droplets that serve as the energy source, were reduced. In addition, 73 lipids, 14 of which were TAGs, were reduced in TOFA-treated Th9 cells alone. We also observed a reduction in the amount of lysophosphatidic acid (LPA), including LPA[14:0] and LPA[16:0], only in *Acaca*^ΔT^ Th9 cells. CS serum Th9 cells alone decreased the levels of 67 types of lipids, most of which included highly saturated fatty acids, including eicosapentaenoic acid (20:5), docosapentaenoic acid (22:5) and docosahexaenoic acid (22:6). As described in Fig. 2H, we observed that PS [16:0/18:1] was commonly decreased in CS-treated, TOFA-treated, and *Acaca*^ΔT^ Th9 cells. In particular, palmitic acid [16:0] and oleic acid [18:0] were the most depleted fatty acids in CS serum (Fig. 2E). The administration of PS [16:0/18:1] moderately suppressed IL-9 production, indicating that a decrease in the amount of PS [16:0/18:1] increased the level of IL-9.

We also observed that changes in FA biosynthesis or the incorporation of extracellular lipids increased the levels of certain intracellular lipid species. Lipidomic analysis revealed that the suppression of FA biosynthesis or FA incorporation caused the accumulation of arachidonic acid (20:4)-containing lipids, such as DAG, phosphatidic acid (PA), phosphatidylcholine (PC), phosphatidylethanolamine (PE), phosphatidylinositol (PI), and cardiolipin (CL), as well as some of the lyso-derivatives of these lipids. There was an increase in the amount of DAG (17:0/20:4), PA (16:0/20:4), PC (19:0/20:4), PE (17:0/20:4), PE (19:0/20:4), PI (17:0/20:4), CL (18:2/18:2/20:4/20:4), LPC (20:4), LPI (20:4), and lysophosphatidylserine (LPS) (20:4). Furthermore, we observed the accumulation of FA (20:3)- or FA (22:6)-containing lipids in TOFA-treated or *Acaca*^ΔT^ Th9 cells but not in CS-treated Th9 cells. In particular, the accumulated lipids, which have 20:3 or 20:6 side chains, include PA, PE, PC, PI, PS, LPC, LPE, LPI, LPG, bis(monoacylglycero)phosphate (BMP), and dilysocardiolipin (DLCL) (Supplementary Table 1). We also focused on the lipid species that were selectively increased under CS serum conditions and observed their accumulation under these conditions. Taken together, these findings suggest that defects in FA biosynthesis tend to increase the degree of FA saturation and decrease the amount of extracellular lipids, causing the accumulation of TAG.

An intriguing finding in the present study is that ACC1-dependent fatty acid biosynthesis negatively regulates IL-9 production in both mouse and human Th9 cells. Previously, we reported that high expression of ACC1 is a hallmark of the production of pathogenic cytokines, including IL-3, IL-5, IL-17, and granulocyte‒macrophage colony‒stimulating factor, by T cells [26, 29]. Given that anabolic fatty acid metabolism is indispensable for membrane biosynthesis and cell growth and proliferation during T-cell activation [17], it may be an exceptional case that de novo fatty acid biosynthesis constrains the IL-9–mediated effector function of T cells. Consistent with our present study, one study has shown that the inhibition of cholesterol metabolism can enhance the differentiation of cytotoxic IL-9-producing cells through the LXR SUMOylation and NF-κB signaling pathways [45]. Notably, cholesterol metabolism is important for the regulation of IL-9 production in CD8^+^ T cells, whereas the fatty acid biosynthesis pathway is critical for the Th9 cell program in CD4^+^ T cells. In our study, palmitic acid, the most common component of saturated fatty acids, and oleic acid, the most common component of monounsaturated fatty acids with reduced expression in *Acaca*^ΔT^ murine Th9 cells, controlled IL-9 production in CD4^+^ Th9 cells. Thus, although dynamic changes in cellular lipid metabolism—including fatty acid and cholesterol metabolism—are essential for the adaptive immune system, which metabolic pathways are major and to what extent they regulate IL-9 production appear to vary among cell types. Further studies focused on elucidating the degree of lipid metabolic dependency of Th9 cell programs in the CD4^+^ and CD8^+^ T-cell lineages will help expand our understanding of the crosstalk between lipid metabolism and acquired immune responses.

Traditionally, the differentiation of different Th cell subsets is thought to depend solely on selective transcription factors that are induced by a variety of specific cytokines and other signaling molecules [46]. However, Th9 cells appear to be an exception, given that all the recognized transcription factors identified thus far are not Th9 cell specific. Instead, many transcription factors are able to facilitate the induction of Th9 cells to various degrees [1]. Indeed, the differentiation of Th9 cells is intricately governed by a multifaceted network of transcription factor modules encompassing cytokine-dependent factors (STATs, SMADs, and TAK1), antigen receptor–dependent factors (BATF/IRF4, NFAT, and NF-κB), and lineage-defining factors (ETV5/PU.1) [47]. In addition to transcription factors, some tumor necrosis factor receptor superfamily members, such as OX40 and GITR, exhibit remarkable potential to promote Th9 cell differentiation through activation of the TRAF6–NF-κB pathway [48]. In this context, our present demonstration of the involvement of fatty acid metabolism in the expression of *Il9* and Th9 cell differentiation is a significant advancement. Our findings revealed that anabolic fatty acid metabolism, which is essential for the differentiation of the majority of Th subsets, instead disturbs the differentiation of Th9 cells. Notably, the inhibition of de novo fatty acid biosynthesis promoted TGF-β–SMAD2/3 pathway signaling. In association with our observations, the inhibition of ACC1 promotes the development of murine Foxp3^+^ regulatory T cells, which are induced by TGF-β–SMAD2/3 pathway signaling, both in vitro and in vivo [49]. Furthermore, the suppression of ACC1 increases epithelial–mesenchymal transition and tumor metastasis by increasing the expression of cellular acetyl-CoA and inducing SMAD2 acetylation in breast cancer cells [50]. Thus, our results, together with those of previous studies, indicate that the inhibition of de novo fatty acid biosynthesis by targeting ACC1 amplifies TGF-β–SMAD signaling.

The RA–RARα axis controls T-cell immune responses and involves both regulatory and inflammatory circuits [33, 51]. In Th9 cells, the RA–RARα axis globally antagonizes Th9-promoting transcription factors and inhibits cell differentiation [33]. Since RARs are recognized for their capacity to selectively interfere with the TGF-β–SMAD signaling pathway in a ligand-specific manner, it is conceivable that they also suppress Th9 cell differentiation *via* a similar pathway [52]. In the present study, we found that RA–RARα signaling is involved in the augmentation of Th9 cell differentiation caused by a restriction of anabolic fatty acid metabolism. It has been reported that saturated and unsaturated fatty acids regulate RA signaling and suppress tumorigenesis *via* fatty acid binding protein 5 (FABP5); saturated fatty acids displace RA from FABP5 and thereby divert the hormone to RARα and activate this receptor [53]. Although we were unable to examine cellular RA due to technical limitations related to the current lipidomics technologies and the tiny amount of RA present in T cells, it is possible that a reduction in the levels of cellular fatty acid metabolites, including palmitic acid or oleic acid, might increase the association of cellular RA with FABP5 and subsequently inhibit RARα activity. Indeed, in the present study, RA administration significantly decreased *Il9* expression and SMAD2/3 nuclear localization in ACC1-inhibited Th9 cells at almost equivalent levels to those in control Th9 cells. Furthermore, palmitic and oleic acids abrogated IL-9 production in TOFA-treated Th9 cells but not in the presence of an RA antagonist. We therefore believe that palmitic and oleic acids synthesized by ACC1 constrain the nuclear translocation of SMAD2/3 and *Il9 via* the activation of RA-RARα signaling, consequently leading to the suppression of Th9 cell differentiation.

Adaptive cell therapy (ACT), such as chimeric antigen receptor T-cell therapy, is effective for the treatment of hematologic malignancies [54]. Although antitumor therapy with Th9 cells has been reported to have superior efficacy compared with therapy with other T-cell subsets [6, 40, 55], acquired resistance is an important mechanism by which tumors escape immune attacks such as those induced by ACT [56]. Thus, one of the issues of ACT is that the transferred immune cells become exhausted early, and it is difficult to maintain them for a long period of time [57]. The present findings show that the inhibition of de novo fatty acid biosynthesis may allow more Th9 cells to stay in the tumor and to more efficiently induce the activation of CTLs. Additionally, we previously reported that the inhibition of ACC1-mediated fatty acid biosynthesis in effector Th cells enhances the generation of memory T cells [27], suggesting that the inhibition of ACC1 in differentiating T cells suppresses exhaustion and potentiates long-term antitumor activity. Another important finding from the present study related to antitumor activity is that the antitumor effect of TOFA-treated Th9 cells was substantially dependent on IL-9. Since IL-9 is known to induce *Ccl20* and *Ccl11*, which contribute to the tissue infiltration of CTLs [40], TOFA-Th9 ACT combined with the transfer of chimeric antigen receptor–expressing CTLs may further enhance the antitumor effect. Furthermore, our in vitro data indicate that deprivation of environmental fatty acids enhances IL-9 production by Th9 cells. The restricted availability of fatty acids is a pivotal characteristic of the metabolic profile of the tumor microenvironment [58, 59]. A recent study also revealed that the solid tumor microenvironment perpetually activates ACC1 in tumor-infiltrating CD8^+^ T cells, leading to lipid biogenesis and storage, which opposes the degradation of fatty acids for energy production [59]. Taken together, these findings suggest that the antitumor activity of ACC1 could promote the potential of Th9 cells, as well as CD8 + T cells, to operate with increased effects therein.

Melanoma patients have fewer Th9 cells in their peripheral blood than healthy controls do [60]. Our publicly available dataset analysis data support the utility of Th9 cells in the prognosis of melanoma patients, as OS and PFS were longer in patients with high expression of Th9-related genes than in patients with low expression.

To conclude, we demonstrate here that reduced levels of intracellular fatty acids augment the differentiation of Th9 cells and that the inhibition of fatty acid biosynthesis in Th9 cells enhances their antitumor activity. Importantly, fatty acids synthesized by ACC1 appear to constrain the nuclear translocation of SMAD2/3 *via* the activation of RA-RARα signaling, as shown by the fact that specific fatty acid species abrogated IL-9 production by TOFA-treated Th9 cells but did not decrease IL-9 in the presence of an RA antagonist. In addition, fatty acids also regulate the binding of RARα to the *Il9* gene locus and suppress *Il9* transcription. Thus, our findings shed light on the mechanisms underlying the connection between fatty acid metabolism and Th9 cell differentiation and antitumor activity.

## Materials and methods

### Mice

*Acaca*^fl/fl^ mice [30] were crossed with CD4-Cre mice (Jackson Laboratory) 10 times and maintained on a C57BL/6 background. Ly5.1 mice (Sankyo Laboratory) were crossed with OT-II-TCR-αβTg mice (Jackson Laboratory). C57BL/6 mice were purchased from Clea Inc., Tokyo, Japan. All mice, male or female, were used at 8–10 weeks of age and were housed under specific-pathogen-free conditions. All of the experiments and animal care were performed in accordance with the guidelines of the Kazusa DNA Research Institute. The animal experiments were performed according to protocols approved by the Institutional Animal Care and Use Committee of the Kazusa DNA Research Institute (Registration number: 30-1-002).

### Antibodies

PE-conjugated anti-CD45.2 (12-0454-82, 104) was purchased from eBioscience. PE-conjugated anti-mouse IL-9 (514104, RM9A4), anti-human IL-9 (507605, MH9A4), APC-conjugated anti-human/mouse granzyme B (372204, QA16A02), anti-human IL-17A (512334, BL168), Alexa647-conjugated anti-mouse IL-17A (506912, TC11-18H10.1), FITC-conjugated anti-mouse CD45.1 (110706, A20), anti-mouse IFNγ (505806, XMG1.2), Alexa488-conjugated anti-mouse Foxp3 (126406, MF-14), BV421-conjugated anti-mouse IL-4 (504120, 11B11), anti-mouse TCR β chain (109230, H57-597), and PE/Cy7-conjugated anti-mouse CD4 (100528, RM4-5) antibodies were purchased from BioLegend (San Diego, CA). Anti-mouse IL-2 (937904), IL-4 (504122), IFNγ (505834), TCR β chain (109254), CD28 (102121), anti-human IFNγ (506532), CD3 (317326), and CD28 (302934) antibodies were also purchased from BioLegend. Alexa647-conjugated anti-human/mouse phospho-Smad2/3 (562696, O72-670), APC-conjugated anti-mouse CD8α (553035, 53-6.7), BV480-conjugated anti-mouse CD44 (566116, IM7), BV605-conjugated anti-mouse TCR β chain (562840, H57-597), and BV786-conjugated anti-mouse CD45 (564225, 30-F11) antibodies were purchased from BD Biosciences. Alexa647-conjugated donkey anti-rabbit IgG (H + L) (A31573) was purchased from Invitrogen. Anti-mouse PD-1 (BE0146), anti-IL-9 (BE0181), and anti-IgG2a (BE0085) antibodies were purchased from Bio X Cell. The anti-Smad2/3 (8685 S) antibody was purchased from Abcam. Anti-H3 (4499 S), anti-Smad2 (5339 S), anti-Smad3 (9523 S), anti-phospho-Smad2 (3108 S), and anti-phospho-Smad3 (9520 S) antibodies were purchased from Cell Signaling.

### Recombinant proteins

Recombinant murine IL-2 (212-12), IL-4 (214-14), IL-12 (200-12), IL-1b (211-11B), IL-6 (216-16), TNFα (315-01 A), granulocyte‒macrophage colony‒stimulating factor (315-03), recombinant human IL-4 (204-IL-010), IL-2 (200-02), and human TGF-β1 (200-01B) were purchased from PeproTech. Recombinant murine IL-23 (1887-ML-010) was purchased from R&D Systems.

### Treatment with fatty acids and lipids

Oleic acid (O1008; Sigma‒Aldrich), palmitic acid (P0500; Sigma‒Aldrich), PE [16:0/18:1] (850757 P: Avanti), or PS [16:0/18:1] (840034 P: Avanti) were dissolved in ethanol to final concentrations of 100 mM (oleic acid), 25 mM (palmitic acid), 10 mM (PE [16:0/18:1] and PS [16:0/18:1]) and complexed with 5% bovine serum albumin prior to use. Docosahexaenoic acid was dissolved in DMSO (1 mM) prior to use. Myristic acid and arachidic acid were obtained from the Fatty Acid Kit (EC10A-1KT; Sigma‒Aldrich) and dissolved in DMSO complexed with 5% bovine serum albumin at a concentration of 100 mM. Palmitoleic acid, linoleic acid, α-linolenic acid and arachidonic acid were obtained from the Fatty Acids Unsaturated Kit (UN10-1KT; Sigma‒Aldrich) and dissolved in DMSO complexed with 5% bovine serum albumin at a concentration of 100 mM. PE [16:0/18:1] and PS [16:0/18:1] were treated with 0.01 mM methyl-beta-cyclodextrin.

### Other reagents and kits

TOFA (T6575; Merck) was dissolved in DMSO (10 mM) prior to use. Curcumin (C7727; Sigma‒Aldrich) was dissolved in ethanol (10 mM) prior to use. Trichostatin A (T8552; Sigma‒Aldrich) was used as a histone deacetylase inhibitor and was dissolved in DMSO (1 mM) prior to use. Retinoic acid (R2625; Sigma‒Aldrich) was dissolved in DMSO (1 mM) prior to use. BMS753 (1084; Sigma‒Aldrich) was used as a RARα agonist and was dissolved in DMSO (1 mM) prior to use. ER 50891 (3823; R&D Systems) was used as a RARα antagonist and was dissolved in DMSO (3 mM) prior to use. C75 (C5490; Sigma‒Aldrich) was used as a fatty acid synthase inhibitor and was dissolved in DMSO (1 mM) prior to use. 5*Z*-7-Oxozeaenol (HY-12686; MCE) was used as a TAK1 inhibitor and was dissolved in DMSO (50 mM) prior to use. A Zombie NIR Fixable Viability Kit (423106) was purchased from BioLegend. Alexidine dihydrochloride (HY-108547; MCE) was used as a PTPMT1 inhibitor and dissolved in DMSO (1 mM) prior to use.

### Mouse cell culture

For the preparation of effector Th cells, naïve CD4^+^ T cells were isolated from the spleens of C57BL/6 J or OTII-TCR-αβTg mice via negative selection via a MojoSort Mouse CD4 T-cell Isolation Kit (BioLegend) and positive selection via CD62L MicroBeads (Miltenyi Biotec). Naïve CD4^+^ T cells were plated onto 24-well tissue culture plates (Costar) precoated with anti-TCRβ (5 mg^/mL^) and anti-CD28 (1 mg^/mL^) antibodies. The cell cultures contained recombinant mouse IL-2 (5 ng^/mL^), an anti-IFNγ antibody (1 mg^/mL^), and an anti-IL-4 antibody (1 mg^/mL^). Th9 cell cultures contained recombinant mouse IL-4 (10 ng mL^–1^), recombinant human TGF-β (1 ng mL^–1^), and anti-IFNγ antibody (1 mg mL^–1^). T cells were cultured with fatty acids at final concentrations of 50 mM (oleic acid), 25 mM (palmitic acid), and 1 mM (docosahexaenoic acid). The following reagents were used at the indicated final concentrations: TOFA (10 mM), RA (1 mM), curcumin (2.5 mM), TSA (3 nM), BMS753 (1 mM), ER 50891 (3 mM), and C75 (1 mM). Th17 cell cultures contained recombinant mouse IL-1β (10 ng mL^–1^), recombinant mouse IL-6 (10 ng mL^–1^), recombinant mouse IL-23 (10 ng mL^–1^), recombinant human TGF-β (1 ng mL^–1^), anti-IL-2 antibody (3 mg mL^–1^), anti-IFNγ antibody (1 mg mL^–1^), and anti-IL-4 antibody (1 mg mL^–1^). After 3 days of culture, differentiated Th0, Th9, or Th17 cells were used in in vitro or in vivo studies.

Mouse bone marrow–derived dendritic cells were generated as described in previous studies [61, 62]. In brief, bone marrow was harvested from femurs and tibiae. Bone marrow cells were filtered through 70 μm nylon mesh (Falcon), and red blood cells were lysed with ACK lysis buffer (A1049201; Gibco). Whole bone marrow cells were plated on 100 mm petri dishes in complete medium supplemented with 10 ng/mL recombinant mouse granulocyte‒macrophage colony‒stimulating factor. Half of the medium was replaced with an equal amount of fresh medium supplemented with granulocyte‒macrophage colony‒stimulating factor on day 4. After 8 days of culture, the medium was replaced with fresh complete medium, and the cells were stimulated with an OVA peptide (Loh15, 5 mM).

### Human blood samples

Human blood samples were obtained from 6 healthy volunteers. A healthy volunteer is an individual who has no known significant health problems and consents to participate in research. The collection of human samples was approved by the local ethics committee and the Review Board of the Kazusa DNA Research Institute. All the volunteers were informed of the usage of their blood samples, and signed consent forms were obtained.

### Human T-cell cultures

Human naïve CD4^+^ T cells were collected with a Naïve CD4^+^ T-Cell Isolation Kit II, human (130–094–131; Miltenyi Biotec) gradient and a Ficoll (17144002; Cytiva) gradient. The T cells were then plated onto 48-well tissue culture plates (Costar) precoated with anti-CD3 (10 mg^/mL^) and anti-CD28 (1 mg^/mL^) antibodies. Th9 cell cultures contained recombinant human IL-4 (30 ng mL^–1^), recombinant human IL-2 (5 ng mL^–1^), recombinant human TGF-β (5 ng mL^–1^), and anti-IFNγ antibody (3 mg mL^–1^).

### Flow cytometry analysis

A FACS Celesta flow cytometer (BD Biosciences) was used. For intracellular cytokine staining, the cells were restimulated with PMA (0.1 mg mL^–1^; Sigma‒Aldrich) and ionomycin (0.5 mM; Merck) plus monensin (Sigma‒Aldrich) for 4 h, followed by fixation and permeabilization. The data were analyzed with FlowJo software (BD Biosciences, v10.8.1).

### qRT‒PCR

Total RNA isolation, cDNA synthesis, and qRT‒PCR were performed as described previously [29] via a StepOnePlus real-time PCR system (Thermo Fisher Scientific) and a TB Green Real-Time PCR kit (TAKARA #RR820A). The primers used were purchased from Thermo Fisher Scientific. Gene expression was normalized to the expression of the housekeeping gene *Hprt* or *18* *S*. The specific primers used are listed in Supplementary Table 3.

### ELISA

ELISA was conducted via a Mouse IL-9 ELISA Kit (442704; BioLegend).

### Immunoblot analysis

Immunoblotting was performed as described previously [29]. In brief, Th9 cells were washed with phosphate-buffered saline (PBS) and lysed with NE-PER Nuclear and Cytoplasmic Extraction Reagents (Thermo Fisher Scientific). After centrifugation for 10 min at 20,*000* *×* *g* and 4 °C to remove debris, the protein concentration in the lysates of the nuclear fraction was measured with Protein Assay Dye Reagent Concentrate (Bio-Rad). The antibodies used for the immunoblot analysis were anti-ACC1 (Cell Signaling Technology), anti-Scd2 (Santa Cruz Biotechnology), and anti-GAPDH (Santa Cruz Biotechnology).

### Lipidome analysis

Untargeted lipid profiling was performed at the Kazusa DNA Research Institute as reported previously with some modifications [28]. In brief, at the end of cell culture, four million cells were spun down, and the pellets were washed with PBS and redissolved in 150 μL of chloroform:methanol (1:2) containing EquiSPLASH (Avanti Polar Lipids) as an internal standard. After sonication for 30 s, 10 μL of water was added, and the mixture was vigorously agitated at 750 rpm for 20 min at 20 °C. The supernatant was collected by centrifugation at *1670* × *g* for 10 min at 20 °C and transferred to a liquid chromatography vial. Liquid chromatography–tandem mass spectrometry analysis was performed via a quadrupole time‒of‒flight mass spectrometer (TripleTOF 6600; Sciex) coupled to an Acquity ultra-performance liquid chromatography system (Waters). Liquid chromatography separation was performed with gradient elution of the mobile phase (A) (methanol/acetonitrile/water [1:1:3, v/v/v] containing 5 mM ammonium acetate [Wako Chemicals] and 10 nM EDTA [Dojindo]) and mobile phase (B) (isopropanol [Wako Chemicals] containing 5 mM ammonium acetate and 10 nM EDTA). The flow rate was 300 μL/min at 45 °C using an L-column3 C18 (50 × 2.0 mm i.d., particle size 2.0 μm; Chemicals Evaluation and Research Institute). The solvent composition started at 100% (A) for the first 1 min and was changed linearly to 64% (B) at 7.5 min, where it was held for 4.5 min; then, the solvent composition was changed linearly to 82.5% (B) at 12.5 min, followed by 85% (B) at 19 min, 95% (B) at 20 min, 100% (A) at 20.1 min, and 100% (A) at 25 min. The raw data files obtained from the analysis were converted to MGF files via the Sciex MS Data Converter program and then used for quantitative analysis with 2DICAL (Mitsui Knowledge Industry). Identification of the molecular species was accomplished by comparison with retention times and tandem mass spectrometry data obtained in information-dependent acquisition mode [63]. Lids containing TOFAs in their structure were omitted from downstream analysis.

### 3′ RNA-seq library preparation

RNA-seq was performed as described previously [28]. In brief, total cellular RNA was extracted with TRIzol reagent (15596-018; Thermo Fisher Scientific). For cDNA library construction, we used a 3′ mRNA library prepared with a QuantSeq 3′ mRNA-Seq Library Prep Kit FWD (015.384; Lexogen). After the PCR step, the size distribution and yield of the library were determined with a D1000 high-sensitivity tape station (5067-5582; Agilent). The pooled libraries were loaded onto a NextSeq 500 system (Illumina) and analyzed by 75-bp single reads.

### Analysis of RNA-seq data

Adaptor sequences were trimmed from the raw RNA-seq reads via the fastp tool (v0.23.1) [64]. The trimmed reads of each sample were mapped to the reference mouse genome mm10 by using the STAR read mapper (v2.3.1) [65] and normalized to 1 million reads in the original library. Genes with an average of 10 or more reads in either group were subjected to further analysis. Genes with at least a 1.5-fold change in expression were defined as differentially expressed genes. Principal component analysis and heatmap construction were conducted with R software (v3.6.0; https://cran.r-project.org/). Gene set enrichment analysis was performed to determine the statistical significance of the enrichment of known transcriptional signatures in a ranked list of genes [33, 66]. Again, principal component analysis and heatmap construction were conducted with R software (v3.6.0), but this time, the amap package (https://CRAN.R-project.org/package=amap) was used.

### ChIP‒qPCR

ChIP qRT‒PCR was performed as described previously [17, 28]. In brief, 5 × 10^6^ Th9 or Th17 cells (cultured for 3 days under Th9- or Th17-skewing conditions) were fixed in 1% paraformaldehyde at 25 °C for 10 min. The lysates were sonicated with an M220 focused ultrasonicator (Covaris). qRT‒PCR was performed on a StepOnePlus real-time PCR system with TB Green Premix Taq II (Takara). The H3-K9 acetylation data for Th9 cells were obtained from the Database of Transcriptional Start Sites (http://dbtss.hgc.jp/). Anti-RARα (62294 S), anti-Smad2 (5339 S), anti-Smad3 (9523 S), and anti-acetyl histone H3-K9 antibodies (Cell Signaling Technology) were used in the ChIP assay. The following specific primers were used: *Il9* gene body FW: 5′-TGATTGTACCACACCGTGCT-3′, RV: 5′-TATCCTTTTCACCCGATGGA-3′; *Il9* CNS2 FW: 5′-TCACCCACTTTAGTCCTTTCAAAA-3′, RV: 5′-AATTACAGAATTTTGCCCCAGGTCCTG-3′; *Il9* CNS25 FW: 5′-ATGTCATGAGGCTTGTCTGC-3′, RV: 5′-ACTCCTAATCTTCAAGCCCCT-3′; *Hprt* promoter FW: 5′-TCCTCCTCAGACCGCTTTT-3′, RV: 5′-TCTGCTGGAGTCCCCTTG-3′.

### ChIP-seq library preparation

The library for ChIP-seq was prepared with a NEBNext Ultra II DNA Library Prep Kit (E7645L; New England Biolabs) for Illumina. After the PCR step, the size distribution and yield of the library were determined with an Agilent High Sensitivity DNA Kit (5067–5583; Agilent) on an Agilent 2100 bioanalyzer. qRT‒PCR was conducted via a GenNext NGS Library Quantification Kit (NLQ-101; Toyobo) to determine the library concentration. The pooled libraries were loaded onto an Illumina NextSeq 500 system and analyzed by 75-bp single reads.

### Analysis of ChIP-seq data

ChIP-seq analysis was performed as described previously [28]. Adaptor sequences were trimmed from the raw ChIP-seq reads with fastp (v0.23.1). The trimmed reads for each sample were mapped to the mm10 reference mouse genome by using Bowtie2 (v0.12.8) [67], and peak calling was performed with MACS2 (v2.1.2) [68]. The generated files were used for constructing a heatmap with DiffBind (v3.15) [69]. BED files recording ChIP-seq signals were converted to BiGWig files by using deepTools (v3.5.0) [70] and normalized to reads per genome coverage. Integrative Genomics Viewer software (v2.4.1) [71] was used for visualization of the BiGWig files. HOMER tag directories were created with “makeTagDirectory” of HOMER software (v4.1.0) [72] from the aligned sequence alignment/map created via SAMtools (v1.15.1) [73]. The tag count density was normalized as tags per 10 million reads in the original library. The “findPeaks” function was used to perform peak calling, and ChIP peaks were annotated to the promoters of the closest genes via HOMER annotatePeaks.pl (mm10 genome build). The “mergePeaks” function was used to obtain control specific, TOFA-treated specific, or common ChIP peaks in Th9 or Th17 cells. The “getDifferentialPeaks” function was used to obtain differentially expressed peaks (fold change > 2.0, *P*-value < 0.0001). These analyses were performed separately in two independent experiments, and finally, the genes commonly altered in two independent experiments were used for analysis via RNA-seq.

### ATAC-seq sample preparation

ATAC-seq samples were prepared as described previously [74]. In brief, 100,000 cells were pelleted and washed with 50 μL of PBS, followed by treatment with 50 μL of lysis buffer. The nuclei were resuspended in a 40 μL transposition reaction with 2 μL of Tn5 transposase (active motif) to tag and fragment the accessible chromatin. The mixture was incubated at 37 °C with shaking at 300 rpm for 30 min. The fragmented DNA was purified via a column from the ATAC-seq kit (Active motif) and amplified with 11 or 12 PCR cycles on the basis of the amplification curve. After the PCR step, the size distribution and yield of the library were determined with an Agilent High Sensitivity DNA Kit (Agilent) on a bioanalyzer. qRT‒PCR was conducted via a GenNext NGS Library Quantification Kit (Toyobo) to determine the library concentration. The pooled libraries were loaded onto a NextSeq 500 (Illumina system) and analyzed by 75-bp single reads.

### Analysis of ATAC-seq data

Adaptor sequences were trimmed from the raw ATAC-seq reads with fastp. Trimmed reads of each sample were mapped to the mm10 reference mouse genome by using Bowtie2 [67]^,^ and peaks were called with MACS2 [68]. The generated files were used for heatmap construction with DiffBind [69]. BED files recording ATAC-seq signals were converted to BiGWig files via deepTools [70] and normalized to reads per genome coverage. Integrative Genomics Viewer software [71] was used for visualization of the BiGWig files. The genome browser files were also viewed with Integrative Genomics Viewer software.

### Immunofluorescence

The cells were fixed with 4% formaldehyde for 10 min, permeabilized with 0.1% Triton X-100 in PBS for 10 min on ice, blocked with 3% bovine serum albumin in PBS for 15 min, and stained with the indicated antibodies for 30 min in the dark. Alexa 647-conjugated goat anti-rat/rabbit IgG F(ab)02 fragments were used as secondary antibodies together with DAPI counterstaining. Images were obtained under a confocal laser microscope (LSM800; Carl Zeiss). For statistical analysis, 3–10 nuclear areas in Th9 cells were examined, and the mean fluorescence was calculated. At least three independent experiments were performed, each yielding similar results.

### Cas9-mediated genome editing

Short guide RNAs were designed by using the online tool provided by CHOPCHOP (http://chopchop.cbu.uib.no) [75]. Freshly isolated splenic CD44^lo^CD62L^hi^ naïve CD4^+^ T cells were activated with plate-bound anti-TCRβ and anti-CD28 antibodies under Th9 cell culture conditions. Cas9 proteins were prepared immediately before the experiments by incubating 1 mg of Cas9 with 0.3 mg of sgRNA in transfection buffer at room temperature for 10 min. After activation for 24 h, T cells were electroporated with a mixture of the Cas9/sgRNA complex via a Neon Transfection Kit and device (Thermo Fisher Scientific). The negative control in the CRISPR Cas9 editing experiments was a nontargeting sgRNA sequence that does not recognize any sequence in the mouse genome. The following sgRNA sequences were used: Smad2-1: AAGCCGTCTACAGTGAGCGAGGG; Smad2-2: AGCAAATACGGTAGATCAGTGGG; Smad3-1: CAGGCGCTTCACGATCGGGGGGG; and Smad3-2: GGCGGCAGTAGATAACGTGAGGG.

### Cell lines

The B16-OVA mouse melanoma cell line was cultured in RPMI 1640 (Thermo) supplemented with 10% heat-inactivated fetal bovine serum (Cytiva). The MC38-OVA mouse colon adenocarcinoma line was cultured in Dulbecco’s modified Eagle’s medium (Gibco) supplemented with 10% heat-inactivated fetal bovine serum (Cytiva) and puromycin (1 mg mL^–1^).

### Tumor models and adoptive transfer

The mice received a subcutaneous abdominal injection of 2 × 10^5^ B16-OVA or MC38-OVA tumor cells. Five days after tumor injection, the mice were subjected to adoptive transfer of 2 × 10^6^ Th9- or TOFA-treated Th9 cells via intravenous injection. For the αPD-1 study, the mice were intraperitoneally administered an αPD-1 antibody (250 mg/mouse) on days 5, 9, and 13.

For the αIL-9 study, the mice received a subcutaneous abdominal injection of 2 × 10^5^ B16-OVA. Cyclophosphamide (Sigma) was administered intraperitoneally as a single dose at 200 mg/kg 1 day before T-cell transfer, as described in a previous study [6]. At 10 days after tumor injection, the mice were subjected to adoptive transfer of 2 × 10^6^ Th9- or TOFA-treated Th9 cells with 2 × 10^5^ peptide-pulsed bone marrow-derived dendritic cells. The mice were intraperitoneally administered an αIL-9 antibody (200 mg/mouse) on days 9, 10, 12, 15, 18, and 21.

### Statistical analysis

The data were analyzed with Prism (v9.4.1; GraphPad) and are expressed as the means ± SDs. Differences were assessed via two-tailed Student’s *t*-tests or two-way ANOVA with Sidak’s multiple comparisons test. In the publicly available dataset analysis, PFS and OS were defined as the time from nivolumab or pembrolizumab administration to the first observation of disease progression or death from any cause and the time from nivolumab administration to death from any cause, respectively. PFS and OS were analyzed via the Kaplan‒Meier method and compared among the groups via the log-rank test. Differences with values of *P* < 0.05 were considered significant. Statistical details are provided in the figure legends.

## Supplementary information

Supplementary Information

Supplementary Figure 1

Supplementary Figure 2

Supplementary Figure 3

Supplementary Figure 4

Supplementary Figure 5

Supplementary Figure 6

Supplementary Figure 7

Supplementary Table 1

Supplementary Table 2

Supplementary Table 3
