# Author Correction: Fatty acid metabolism constrains Th9 cell differentiation and antitumor immunity *via* the modulation of retinoic acid receptor signaling

**DOI:** 10.1038/s41423-024-01223-0

**Published:** 2024-10-08

**Authors:** Takahiro Nakajima, Toshio Kanno, Yuki Ueda, Keisuke Miyako, Takeru Endo, Souta Yoshida, Satoru Yokoyama, Hikari K. Asou, Kazuko Yamada, Kazutaka Ikeda, Yosuke Togashi, Yusuke Endo

**Affiliations:** 1 Department of Frontier Research and Development, Laboratory of Medical Omics Research, Kazusa DNA Research Institute, 2-6-7 Kazusa Kamatari, Kisarazu, Chiba 292-0818 Japan; 2 Department of Tumor Microenvironment, Faculty of Medicine, Dentistry and Pharmaceutical Sciences, Okayama University, 2-5-1, Shikata-cho, Kita-ku, Okayama 700-8558 Japan; 3 Department of Applied Genomics, Kazusa DNA Research Institute, 2-6-7 Kazusa Kamatari, Kisarazu, Chiba 292-0818 Japan; 4 Division of Cell Therapy, Chiba Cancer Center Research Institute, 666-2 Nitona-cho, Chuo-ku, Chiba 260-8717 Japan; 5 Department of Omics Medicine, Graduate School of Medicine, Chiba University, 1-8-1 Inohana, Chuo-ku, Chiba 260-8670 Japan

**Keywords:** Interleukins, Tumour immunology, Lipid signalling

Correction to: *Cellular & Molecular Immunology* 10.1038/s41423-024-01209-y, published online 26 August 2024

The data availability statement was mistakenly omitted in the published article. The statement should read as follows:

“The RNA-seq, ATAC-seq, and ChIP-seq data have been deposited in the Gene Expression Omnibus at NCBI (https://www.ncbi.nlm.nih.gov/) under accession number GSE275438.”

