# Supplementary material for: Fatty acid metabolism constrains Th9 cell differentiation and antitumor immunity via the modulation of retinoic acid receptor signaling
Source: Cell Mol Immunol. 2024 Aug 26;21(11):1266–81. doi: 10.1038/s41423-024-01209-y (PMC11528006; doi:10.1038/s41423-024-01209-y)
Supplement: Supplementary file 1 — Supplementary Information [file 41423_2024_1209_MOESM1_ESM.docx]

**Supplementary Fig. 1 Expression of fatty acid uptake, fatty acid biosynthesis, and Th9 related genes in T cell subsets**

**(a)** Quantitative RT-PCR analysis of fatty acid uptake related genes, including *Ldlr, Lrp8, Scarb1 and Vldlr*, in naïve CD4^+^, Th0 and Th9 cells. **(b)** Quantitative RT-PCR analysis of *Batf3* and *Irf4* in Th9 cells under charcoal-stripping (CS) condition. **(c)** Quantitative RT-PCR analysis of *BATF3 IRF4* and *SPI1* in human Th9 cells under CS condition. **(d)** Quantitative RT-PCR analysis of de novo fatty acid biosynthesis related genes, including *Acaca, Scd1, Scd2, Acsl3, Elovl1, Elovl5, and Fads2*, in naïve CD4^+^, Th0 and Th9 cells. **(e)** Microscopy analysis was performed using naïve CD4^+^, Th0 and Th9 cells stained with lipid droplet and nuclear. The scale bars represent 5 μm.

**(f)** Quantitative RT-PCR analysis of *Batf3* and *Irf4* in Th9 and TOFA-Th9 cells. **(g)** Quantitative RT-PCR analysis of *BATF3 IRF4* and *SPI1* in human Th9 and TOFA-Th9 cells. *n* =3 (e); 4 (a), (b), (d) and (f); 6 (c) and (g) per each group biologically independent samples are shown. More than three independent experiments were performed with similar results for (a)-(g). Mean values with s.d. are shown for (a)-(d), (f) and (g). An unpaired two-tailed student’s t-test was applied for (a)-(d), (f) and (g). Statistical significances (*P* value) are indicated as **P* < 0.05; ***P* < 0.01; ****P* < 0.001; *****P* < 0.0001; N.S. = not significant.

**Supplementary Fig. 2 Expression of fatty acid biosynthesis related genes in *Acaca*^ΔT^ Th9 cells.**

**(a)** Quantitative RT-PCR analysis of *Batf3* and *Irf4* in Th9 cells. The cells are collected from *Acaca*^fl/fl^ or *Acaca*^ΔT^ mice, and cultured under Th9 condition for 3 days in vitro. **(b)** Venn diagram depicting the number of increased lipids in CS-serum, TOFA-treated and *Acaca*^ΔT^ Th9 cells as compared with control cells. **(c, d)** Representative intracellular staining profiles of IL-9 and IL-17A in Th9 cells with vehicle, stearic acid (c), or vehicle, myristic acid, palmitoleic acid, linoleic acid, α-linolenic acid, arachidic acid, or arachidonic acid (**d**) in the presence of TOFA. **(e, f)** Representative intracellular staining profiles of IL-9 and IL-17A in Th9 cells with vehicle, DHA (e) or PTPMT1-specific inhibitor alexidine dihydrochloride (AD) (f). The cells are collected from *Acaca*^fl/fl^ or *Acaca*^ΔT^ mice, and cultured as in (a). More than three independent experiments were performed with similar results for (a) and (c-f). Lipidomics analysis included three biologically independent samples in each group. Mean values with s.d. are shown for (a) and (c, d). An unpaired two-tailed student’s t-test was applied for (a) and (c). Statistical significances (*P* value) are indicated as **P* < 0.05; ***P* < 0.01; ****P* < 0.001; *****P* < 0.0001; N.S. = not significant.

**Supplementary Fig. 3 ACC1 also represses permissive chromatin landscape at the *Il9* gene locus in Th17 cells.**

**(a)** Representative intracellular staining profiles of IL-9 and IL-17A in Th17 cells treated with or without TOFA. **(b)** PCA plot of gene expression profiles by RNA-sequencing, including control, TOFA-treated Th17 cells. **(c)** A clustering heatmap depict the gene in control or TOFA-treated Th17 cells. **(d)** MA plot analysis of RNA-seq data of control and TOFA-Th17 cells. Red dots indicate genes involved in retinoic acid-related genes. The set of genes increased more than 2-fold in RARα KO Th9 was defined from a previously published data set (GSE123501). **(e)** Expression profile by RNA-sequencing of Th9-related genes in control, TOFA treated and *Acaca*^ΔT^ Th9 cells. **(f)** Representative intracellular staining profiles of IL-9 and IL-17A in control and TOFA Th9 cells with or without sg*Batf3* transduction. **(g, h)** Venn diagram showed overlaps and differences in peaks between control and TOFA-treated Th9 (g, n=2) or Th17 cells (h, n=1 and 2) by ChIP-seq. **(i)** Average plots and heat maps showing the H3K9ac enrichment in Th17 cells at the TSS in the ChIP-seq data sets. **(j, k)** Western blot analysis of Total H3, H3K27Ac (j), or H3K9me3 (k) from Th9 cells was performed. **(l, m)** Venn diagram showed overlaps and differences in peaks between control and TOFA-treated Th9 by H3K27Ac (l) or H3K9me3 (m) ChIP-seq. Two biologically independent experiments were performed with (b)-(e), (g)-(m). *n* = 1 (g)-(i), (j)-(m); 3 (b)-(f); 4 (a) per each group biologically independent samples are shown. Two biologically independent experiments were performed with (b)-(m). More than three independent experiments were performed with similar results for (a). Mean values with s.d. are shown for (a). An unpaired two-tailed student’s t-test was applied for (a). Statistical significances (*P* value) are indicated as **P* < 0.05; ***P* < 0.01; ****P* < 0.001; *****P* < 0.0001; N.S. = not significant.

**Supplementary Fig. 4 TGFb-Smad2/3 pathway mainly affects TOFA-Th9 enhancement of IL-9 production.**

**(a)** Lists showed; number of control-specific or TOFA-specific peaks (ChIP-seq; H3K9Ac) in Th9 (Fig. 3d and Supplementary Fig. 3g) and Th17 (Supplementary Fig. 3h) cells. **(b)** Venn diagram showed overlaps and differences between 2.0-fold increased genes (RNA-seq) or peaks (ChIP-seq; H3K9ac) in TOFA-treated Th17 cells relative to control Th9 cells. **(c)** Commonly upregulated genes in RNA-seq and ChIP-seq related to Supplementary Fig. 4a are shown here. Left columns, expression; Middle columns, histone acetylation; Right columns, permissive chromatin landscape in TOFA/WT ratio. **(d)** The graph showed IL-9 production in Th9 cells cultured at the indicated concentrations of IL-4 analyzed by FACS. **(e)** Representative intracellular staining profiles of IL-9 and Foxp3 in Th9 cells cultured at the indicated concentrations of IL-4. **(f)** Representative intracellular staining profiles of IL-9 and IL-4 in CD4^+^ T cells at the indicated concentrations of IL-4. **(g)** Representative intracellular staining profiles of IL-9 and IFNγ (Th1), IL-9 and IL-17A (Th9 and Th17), or IL-9 and Foxp3 (Treg). **(h)** Representative intracellular staining profiles of IL-9 in Th9 or TOFA-Th9 cells treated with or without TAK1 inhibitor. *n* = 4 (d)-(h) per each group biologically independent samples are shown. More than three independent experiments were performed with similar results for (d)-(h). Mean values with s.d. are shown for (d) and (h). An unpaired two-tailed student’s t-test was applied for (d) and (h). Statistical significances (*P* value) are indicated as **P* < 0.05; ***P* < 0.01; ****P* < 0.001; *****P* < 0.0001; N.S. = not significant.

**Supplementary Fig. 5 ACC1-mediated fatty acid biosynthesis controls expression of retinoic acid regulatory genes in Th17 cells.**

**(a)** Gene set enrichment analysis (GSEA) revealed decreased expression of genes involved in retinoic acid-related genes in Th17 cells upon treatment with TOFA. **(b)** Heatmap depicting genes differentially expressed in the retinoic acid regulatory genes in control and in TOFA-treated Th17 cells. **(c-d)** Western blot analysis of pSmad2 (c) and pSmad3 (d) in control, TOFA-treated, and TOFA plus retinoic acid-treated Th9 cells. **(e)** ChIP assays were performed with anti-RARα antibody at the *Il9* locus from control, TOFA-treated, and retinoic acid-treated Th9 cells. The intensities of these modifications relative to input DNA were determined by quantitative RT-PCR analysis. *n* = 1 (c); 3 (a) and (b) per each group biologically independent samples are shown. Two independent experiments were performed with similar results for (a), (b) and (e). Three independent experiments were performed with similar results for (c) and (d). An unpaired two-tailed student’s t-test was applied for (e). Statistical significances (*P* value) are indicated as **P* < 0.05; ***P* < 0.01; ****P* < 0.001; *****P* < 0.0001; N.S. = not significant.

**Supplementary Fig. 6 Tumor-specific TOFA-Th9 or combination therapy of αPD-1 and TOFA-Th9 cells treatment remarkably shrinks tumors.**

**(a)** Experimental protocol for the B16-OVA tumor model with OT-II Th9 or TOFA-Th9 cells injection. **(b)** Images of tumors show repressed tumor growth in Th9 or TOFA-Th9 injection groups in the B16-OVA tumor model. **(c)** The number of Granzyme B or IFNγ-producing tumor infiltrated CD8^+^ T cells per mg of tumor in the B16-OVA tumor model. **(d)** The number of tumor infiltrated CD4^+^ T cells per mg of tumor in the B16-OVA tumor model. **(e)** The proportion of CD45.1^+^ transferred Th9 cells in tumor infiltrated CD4^+^ T cells in the B16-OVA tumor model. **(f)** Experimental protocol for the MC38 tumor model with OT-II Th9 or TOFA-Th9 cells injection. **(g)** Images of tumors show repressed tumor growth in Th9 or TOFA-Th9 injection groups in the MC38 tumor model. **(h)** The number of Granzyme B or IFNγ-producing tumor infiltrated CD8^+^ T cells per mg of tumor in the MC38 tumor model. **(i)** The number of tumor infiltrated CD4^+^ T cells per mg of tumor in the MC38 tumor model. **(j)** Experimental protocol for the B16-OVA tumor model with combination therapy of OT-II Th9 or TOFA-Th9 cells injection with anti-PD-1 antibody. **(k)** Individual tumor responses to combination therapy of OT-II Th9 or TOFA-Th9 cell transfer and anti-PD-1 antibody in the B16 tumor model are shown. **(l)** Images of tumors in combination therapy of OT-II Th9 or TOFA-Th9 cell transfer and anti-PD-1 antibody in the B16 tumor model. **(m)** Experimental protocol for the B16 tumor model with OT-II Th9 or TOFA-Th9 cells plus anti-IL-9 antibody injection. **(n)** Individual tumor responses to OT-II Th9 cell transfer with or without anti-IL-9 antibody treatment in the B16 tumor model are shown. **(o)** The survival rate of tumor-bearing mice with indicated treatments. *n* = 4 (b)-(e); 4-5 (g)-(i); 5 (k)-(o) per each group biologically independent samples are shown. More than three independent experiments were performed with similar results for (b)-(e), (g)-(i), (k)-(l), (n) and (o). Mean values with s.d. are shown for (c)-(e), (h)- and (i). An unpaired two-tailed student’s t-test was applied for (c)-(e), (h) and (i). Statistical significances (*P* value) are indicated as **P* < 0.05; ***P* < 0.01; ****P* < 0.001; *****P* < 0.0001; N.S. = not significant.

**Supplementary Fig. 7 Fatty acid regulates IL-9 production *via* Smad2/3 and RARα signaling pathways.**

**(a)** Left: Extrinsic or intrinsic oleic acid/palmitic acid regulate RARα activity that inhibit Smad2/3 phosphorylation and *Il9* transcription. Right: Blockade of ACC1-mediated *de novo* fatty acid biosynthesis suppresses RARα activity, which augments phosphorylation of Smad2/3 and nuclear translocation. In addition, ACC1 inhibition disturbs RARα binding to *Il9* gene locus. Increased nuclear translocation of Smad2/3 and reduced RARα binding to *Il9* gene locus result in a dramatic increase of IL-9 production. (**b**) Upper left: IL-9 produced by Th9 cells augments infiltration of CD8^+^ T cells into the tumor microenvironment. Infiltrated CD8^+^ T cells produce granzyme B and IFNγ, thereby eliminating the tumor. Upper right: TOFA treatment augments Th9 cells to infiltrate into tumor microenvironment and promote IL-9 production, enhancing the proliferation and tumor infiltration of CD8^+^ T cells. Tumors can be efficiently eliminated by the high infiltration of Granzyme B- and IFNγ-producing CD8^+^ T cells. Lower left: Administration of anti-PD-1 antibody with control Th9 cell transfer has little effect on tumor elimination. Lower right: Administration of anti-PD-1 antibody augmented the effects of TOFA-mediated and Th9 cell-dependent tumor elimination.
