# Supplementary figures and images for: Fatty acid metabolism constrains Th9 cell differentiation and antitumor immunity via the modulation of retinoic acid receptor signaling
Source: Cell Mol Immunol. 2024 Aug 26;21(11):1266–81. doi: 10.1038/s41423-024-01209-y (PMC11528006; doi:10.1038/s41423-024-01209-y)

Supplementary Fig. 1

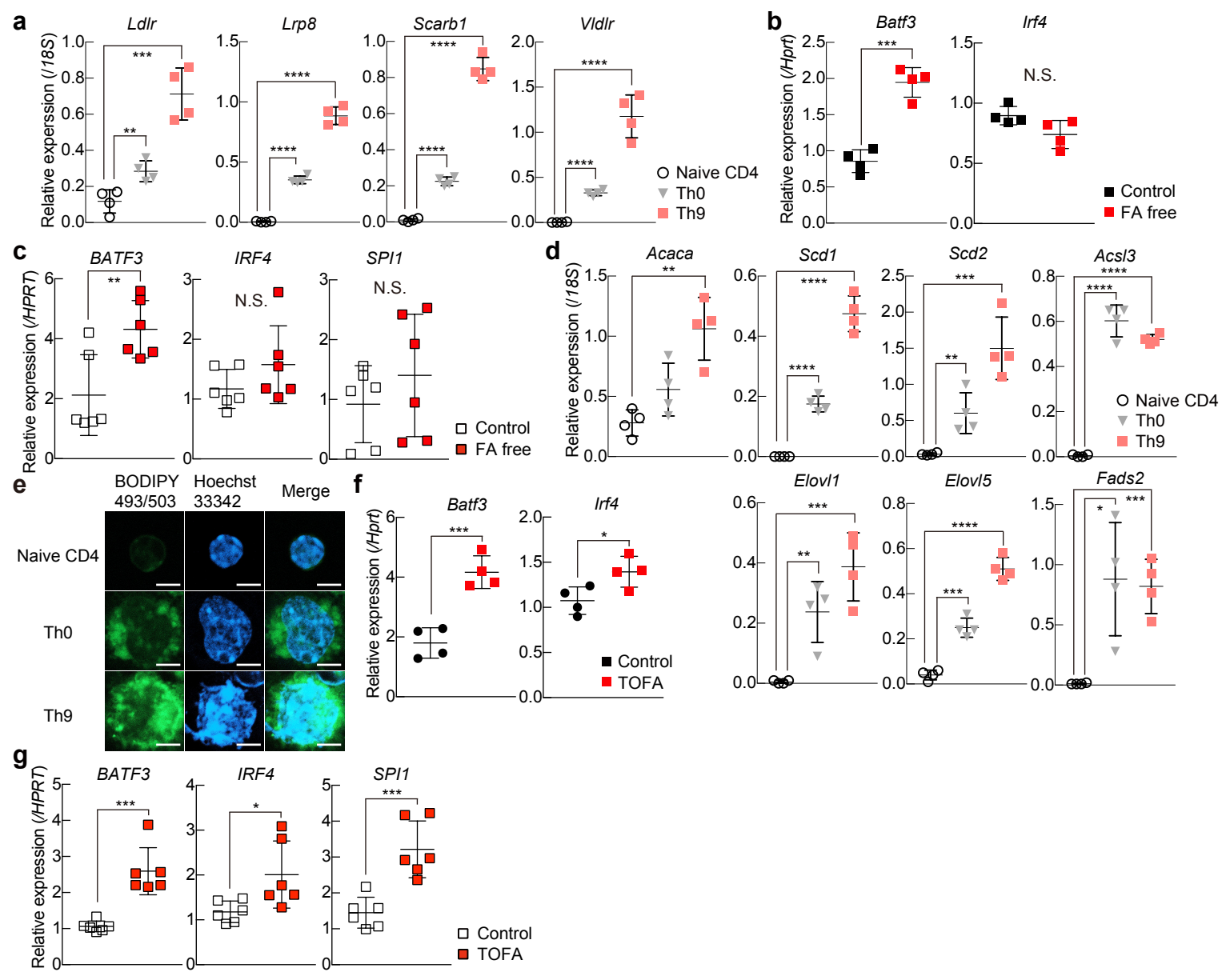

Supplement: Supplementary file 2 — Supplementary Figure 1 [file 41423_2024_1209_MOESM2_ESM.pdf]

Supplementary Fig. 2

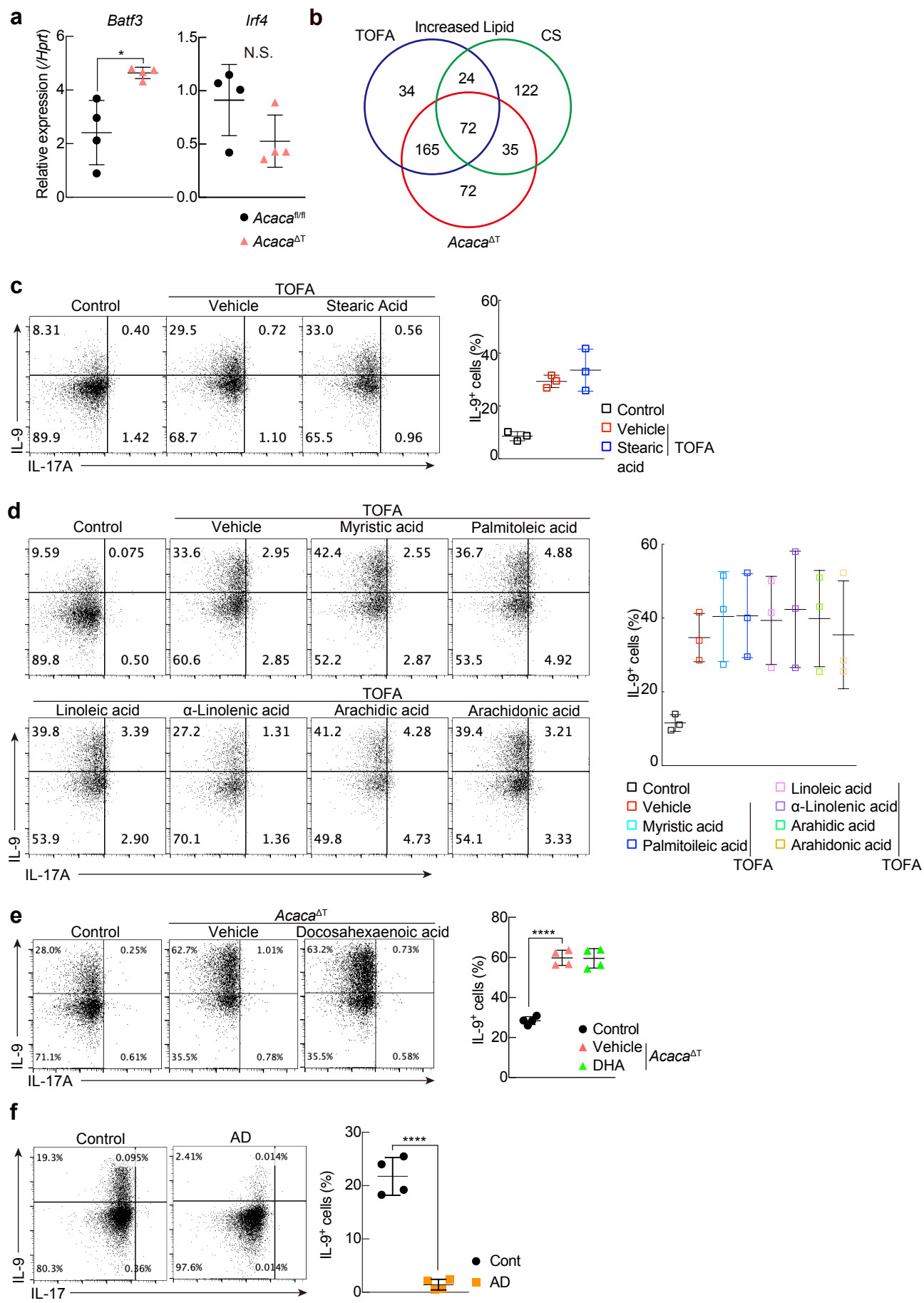

Supplement: Supplementary file 3 — Supplementary Figure 2 [file 41423_2024_1209_MOESM3_ESM.pdf]

**Supplementary Fig. 3**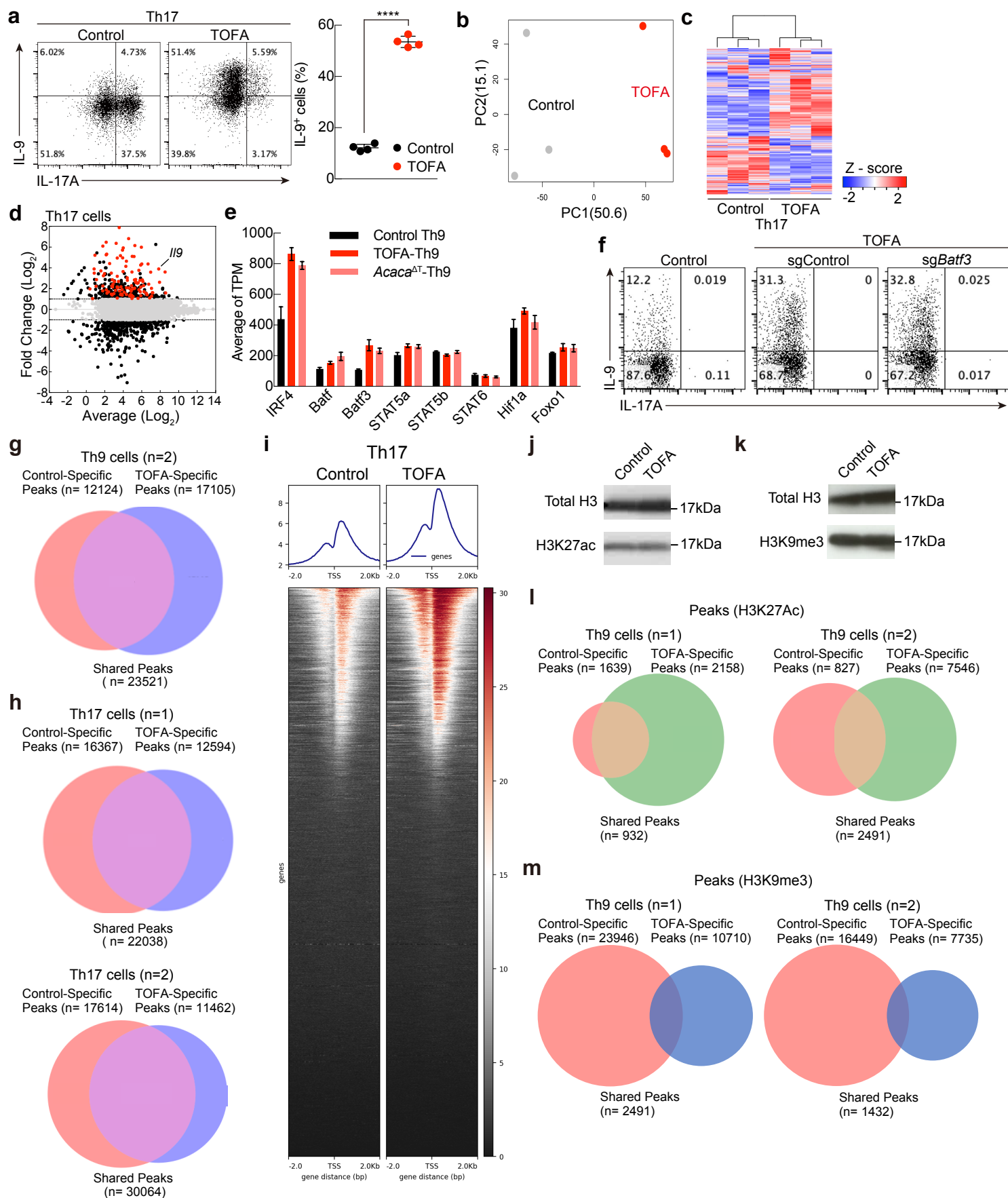

Supplement: Supplementary file 4 — Supplementary Figure 3 [file 41423_2024_1209_MOESM4_ESM.pdf]

**Supplementary Fig. 5**

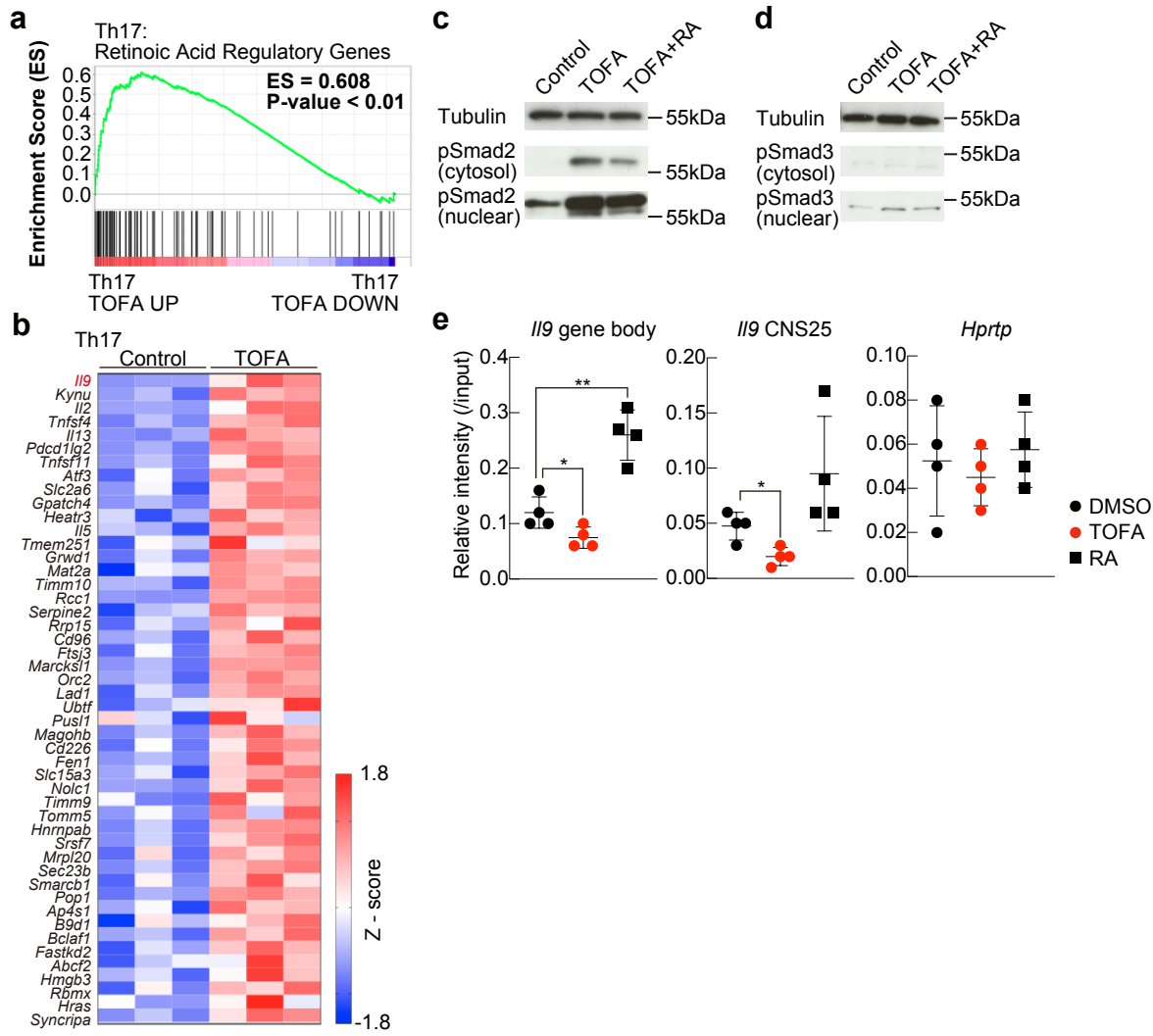

Supplement: Supplementary file 6 — Supplementary Figure 5 [file 41423_2024_1209_MOESM6_ESM.pdf]

**Supplementary Fig. 6**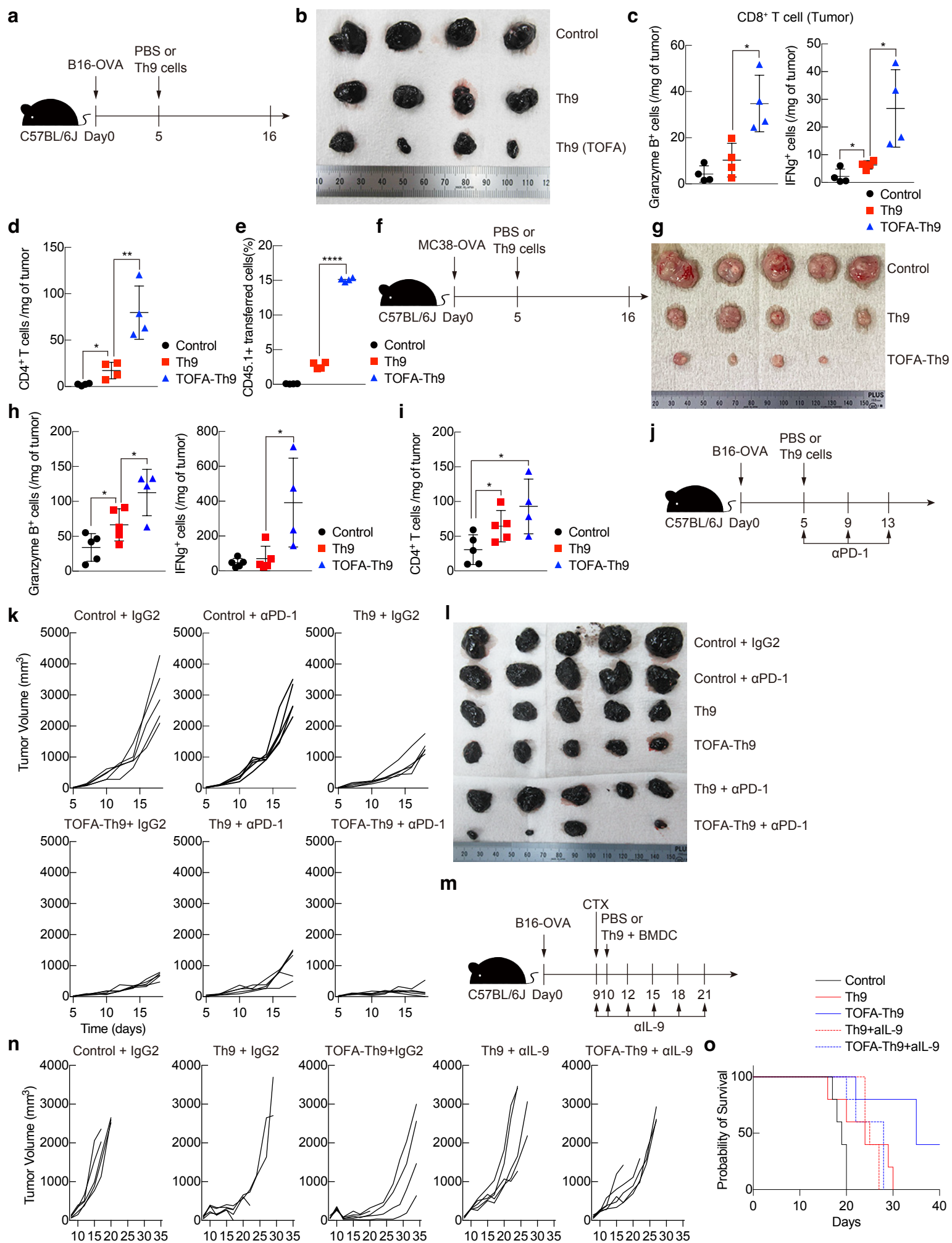

Supplement: Supplementary file 7 — Supplementary Figure 6 [file 41423_2024_1209_MOESM7_ESM.pdf]

**Supplementary Fig. 7**

**a**

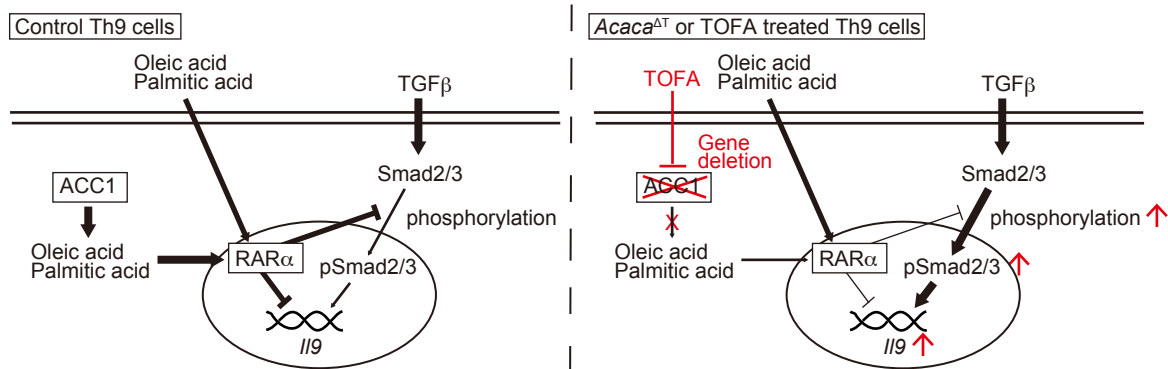

**b**

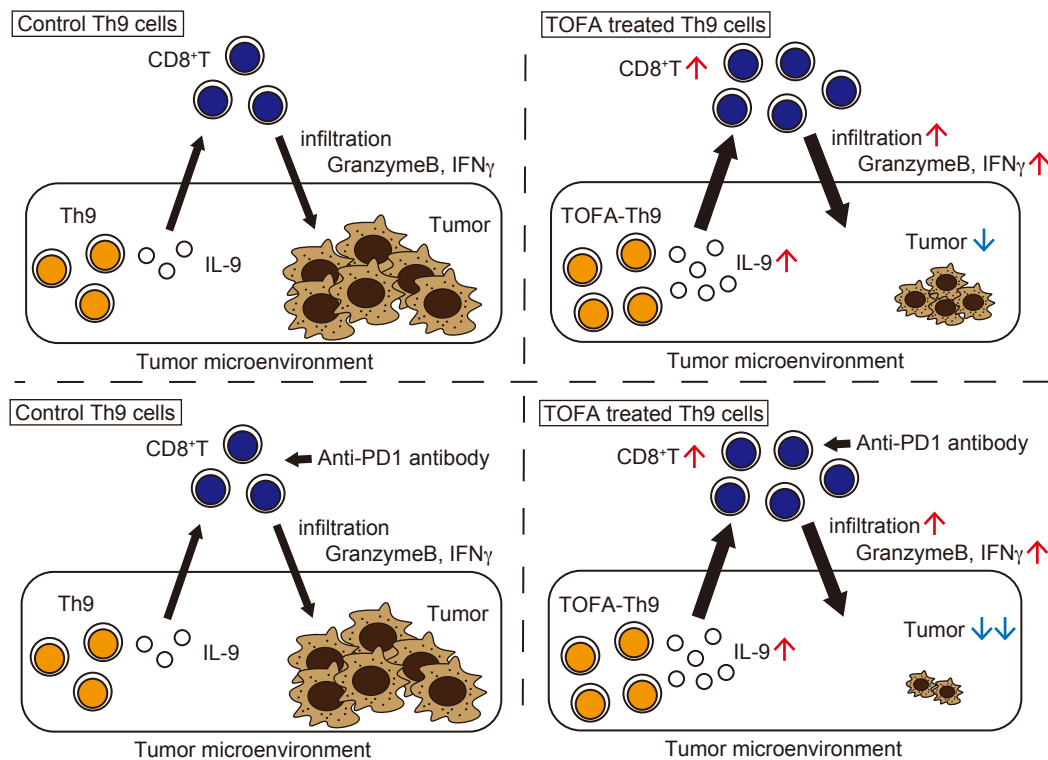

Supplement: Supplementary file 8 — Supplementary Figure 7 [file 41423_2024_1209_MOESM8_ESM.pdf]
