# Supplementary material for: Fatty acid metabolism constrains Th9 cell differentiation and antitumor immunity via the modulation of retinoic acid receptor signaling
Source: Cell Mol Immunol. 2024 Aug 26;21(11):1266–81. doi: 10.1038/s41423-024-01209-y (PMC11528006; doi:10.1038/s41423-024-01209-y)

Supplementary Fig. 4

a

H3K9Ac peaks

Control-specific
Th9 : 15069 (n=1), 12124 (n=2)
Th17 : 17614 (n=1), 16367 (n=2)
TOFA-specific
Th9 : 15478 (n=1), 17105 (n=2)
Th17 : 11462 (n=1), 12594 (n=2)

Differentially changed peaks

TOFA-increased
Th9 : 1915 (n=1), 2019 (n=2)
Th17 : 1429 (n=1), 2407 (n=2)
TOFA-decreased
Th9 : 1648 (n=1), 1020 (n=2)
Th17 : 2301 (n=1), 915 (n=2)

Differentially changed genes

TOFA-increased
Th9 : 1576 (n=1), 1715 (n=2)
Th17 : 1222 (n=1), 1883 (n=2)
TOFA-decreased
Th9 : 1074 (n=1), 766 (n=2)
Th17 : 1581 (n=1), 693 (n=2)

Common genes

TOFA-increased
Th9 : 489
Th17 : 461
TOFA-decreased
Th9 : 188
Th17 : 168

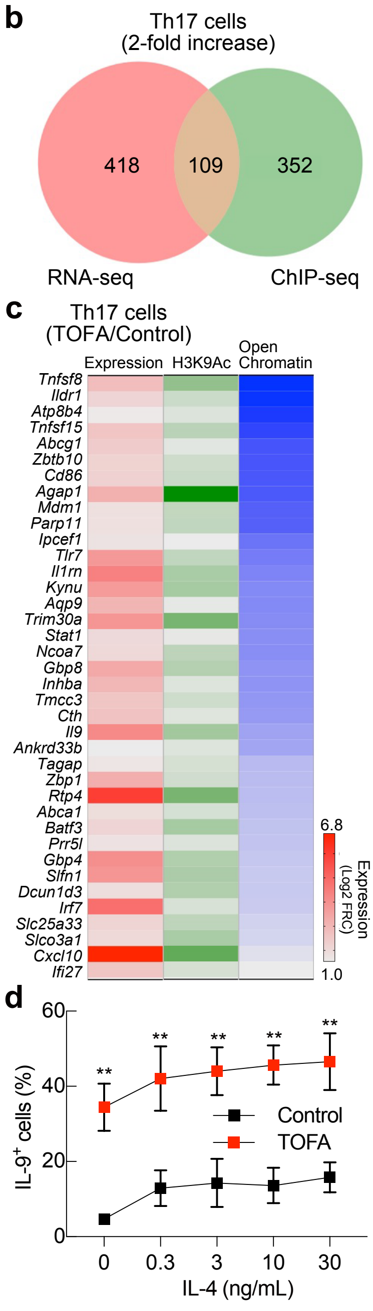

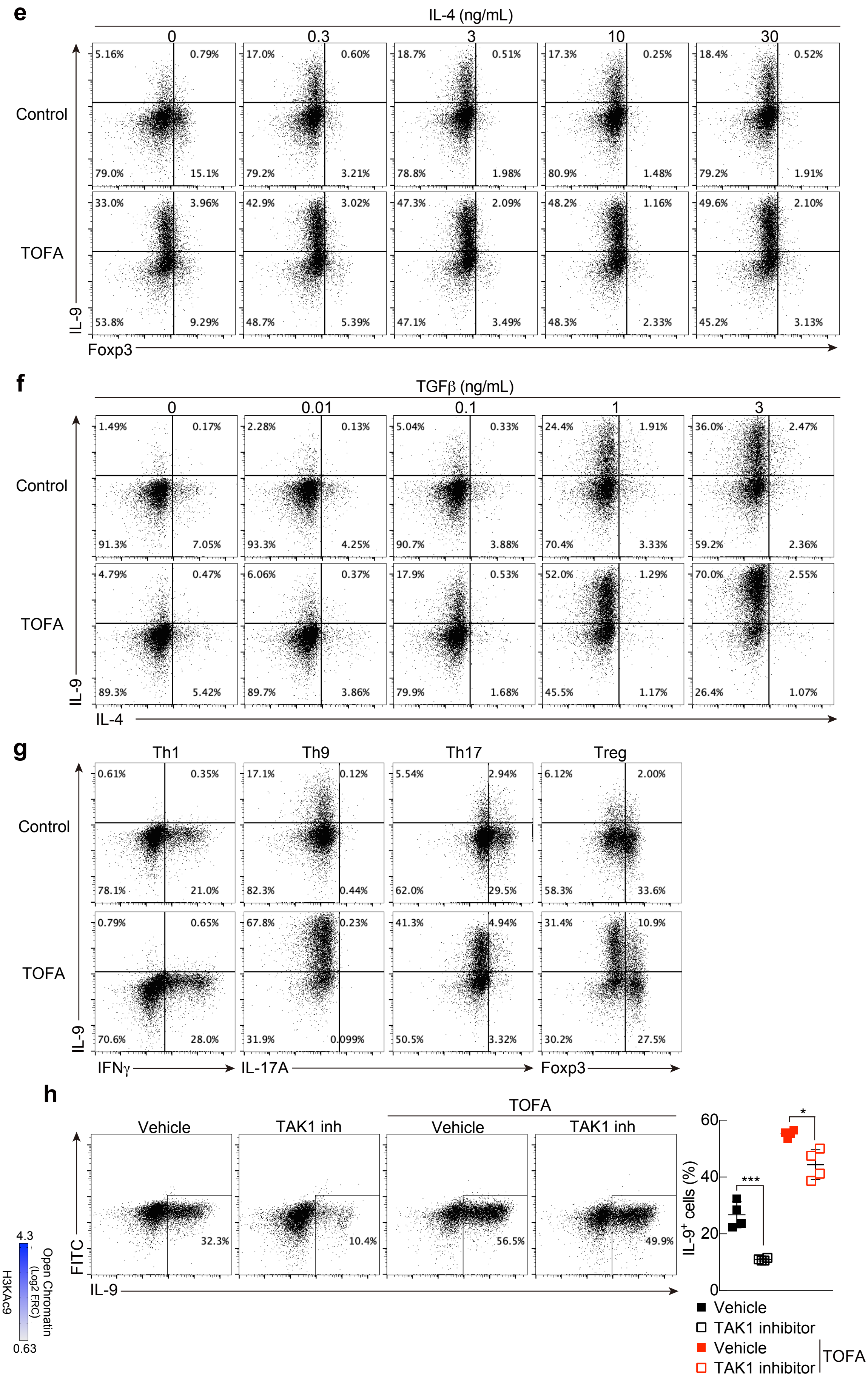

Supplement: Supplementary file 5 — Supplementary Figure 4 [file 41423_2024_1209_MOESM5_ESM.pdf]
